# Genome-wide RNA-seq of iPSC-derived motor neurons indicates selective cytoskeletal perturbation in Brown–Vialetto disease that is partially rescued by riboflavin

**DOI:** 10.1038/srep46271

**Published:** 2017-04-06

**Authors:** Federica Rizzo, Agnese Ramirez, Claudia Compagnucci, Sabrina Salani, Valentina Melzi, Andreina Bordoni, Francesco Fortunato, Alessia Niceforo, Nereo Bresolin, Giacomo P. Comi, Enrico Bertini, Monica Nizzardo, Stefania Corti

**Affiliations:** 1 Dino Ferrari Centre, Neuroscience Section, Department of Pathophysiology and Transplantation (DEPT), University of Milan, Neurology Unit, IRCCS Foundation Ca’ Granda Ospedale Maggiore Policlinico, Milan, Italy; 2 Unit of Neuromuscular and Neurodegenerative Disorders, Laboratory of Molecular Medicine, Bambino Gesu’ Children’s Research Hospital, Rome, Italy

## Abstract

Riboflavin is essential in numerous cellular oxidation/reduction reactions but is not synthesized by mammalian cells. Riboflavin absorption occurs through the human riboflavin transporters RFVT1 and RFVT3 in the intestine and RFVT2 in the brain. Mutations in these genes are causative for the Brown–Vialetto–Van Laere (BVVL), childhood-onset syndrome characterized by a variety of cranial nerve palsies as well as by spinal cord motor neuron (MN) degeneration. Why mutations in RFVTs result in a neural cell–selective disorder is unclear. As a novel tool to gain insights into the pathomechanisms underlying the disease, we generated MNs from induced pluripotent stem cells (iPSCs) derived from BVVL patients as an *in vitro* disease model. BVVL-MNs explained a reduction in axon elongation, partially improved by riboflavin supplementation. RNA sequencing profiles and protein studies of the cytoskeletal structures showed a perturbation in the neurofilament composition in BVVL-MNs. Furthermore, exploring the autophagy–lysosome pathway, we observed a reduced autophagic/mitophagic flux in patient MNs. These features represent emerging pathogenetic mechanisms in BVVL-associated neurodegeneration, partially rescued by riboflavin supplementation. Our data showed that this therapeutic strategy could have some limits in rescuing all of the disease features, suggesting the need to develop complementary novel therapeutic strategies.

Riboflavin (7,8-dimethyl-10-ribityl-isoalloxazine; vitamin B2) is a water-soluble group B vitamin and the precursor of the coenzymes flavin adenine dinucleotide (FAD) and flavin mononucleotide (FMN)^1^. They are essential cofactors in different metabolic processes, including carbohydrate, amino acid, and lipid metabolism and the electron transport chain^2^. Riboflavin cannot be readily synthesized in mammalian cells, so it must be absorbed from the diet via riboflavin transporters (RFVTs)^3^,^4^,^5^. RFVT1 and RFVT3 are predominantly expressed in the intestine and RFVT2 in the brain. The absence of riboflavin in the diet causes a range of developmental and growth disorders^6^. Furthermore, riboflavin supplementation is useful in the treatment of inborn errors of metabolism, such as mild multiple acyl-CoA dehydrogenation defect (MADD) and some mitochondrial diseases^7^,^8^.

In 2010, it was established that autosomal recessive mutations in the riboflavin transporter genes *SLC52A2* (coding for *RFT3, RFVT2*) and *SLC52A3* (alias *C20orf54*, coding for *RFT2, RFVT3*) are responsible for the neurodegenerative disorder previously identified as Brown–Vialetto–Van Laere (BVVL) or Fazio Londe (FL) syndrome^9^,^10^,^11^,^12^,^13^,^14^. *RFVT1* does not seem to be associated with a human disease (or as an alternative lethal at embryonic stage), as no patients with this deficiency have been described.

BVVL syndrome is a rare neurodegenerative disease characterized by progressive ponto-bulbar palsy, bilateral sensorineural hearing loss, muscle weakness, and amyotrophy due to motor neuron (MN) degeneration^13^,^14^,^15^. The principal cause of death is respiratory failure due to diaphragm denervation^9^,^10^,^11^,^12^,^16^,^17^,^18^,^19^, and the FL clinical presentation is similar to BVVL, without the hearing loss. Therapy with high-dose oral riboflavin (B2) was efficacious in BVVL cases with significant clinical improvement^1^. Despite this advancement in the knowledge of riboflavin deficiency disorders, the causal relationship between riboflavin transporter mutations and neural/MN degeneration remains unknown. Addressing this question might contribute not only to increasing understanding of the disease but also to developing novel and more effective treatments for use in association with riboflavin replacement therapy.

In this study, we thus investigated several pathophysiological aspects in induced pluripotent (iPSC)-derived MNs from two BVVL patients (BVVL-MNs). Establishing *in vitro* patient-specific models allowed the identification of MN pathological features. Patient MNs exhibited a different transcriptional signature with respect to wild-type (WT) MNs (WT-MNs), as demonstrated by RNA sequencing analysis, suggesting impairment in cytoskeletal structure, axonogenesis, and neurotrophic factor metabolism. Indeed, we demonstrated a perturbation in mitochondrial function and in the autophagic pathway. These features represent emerging pathogenetic mechanisms in BVVL-associated neurodegeneration and possible targets for therapeutic intervention, in addition to riboflavin supplementation.

## Results

### Axonal elongation was significantly affected in MNs derived from BVVL patients

**Figure 1:**
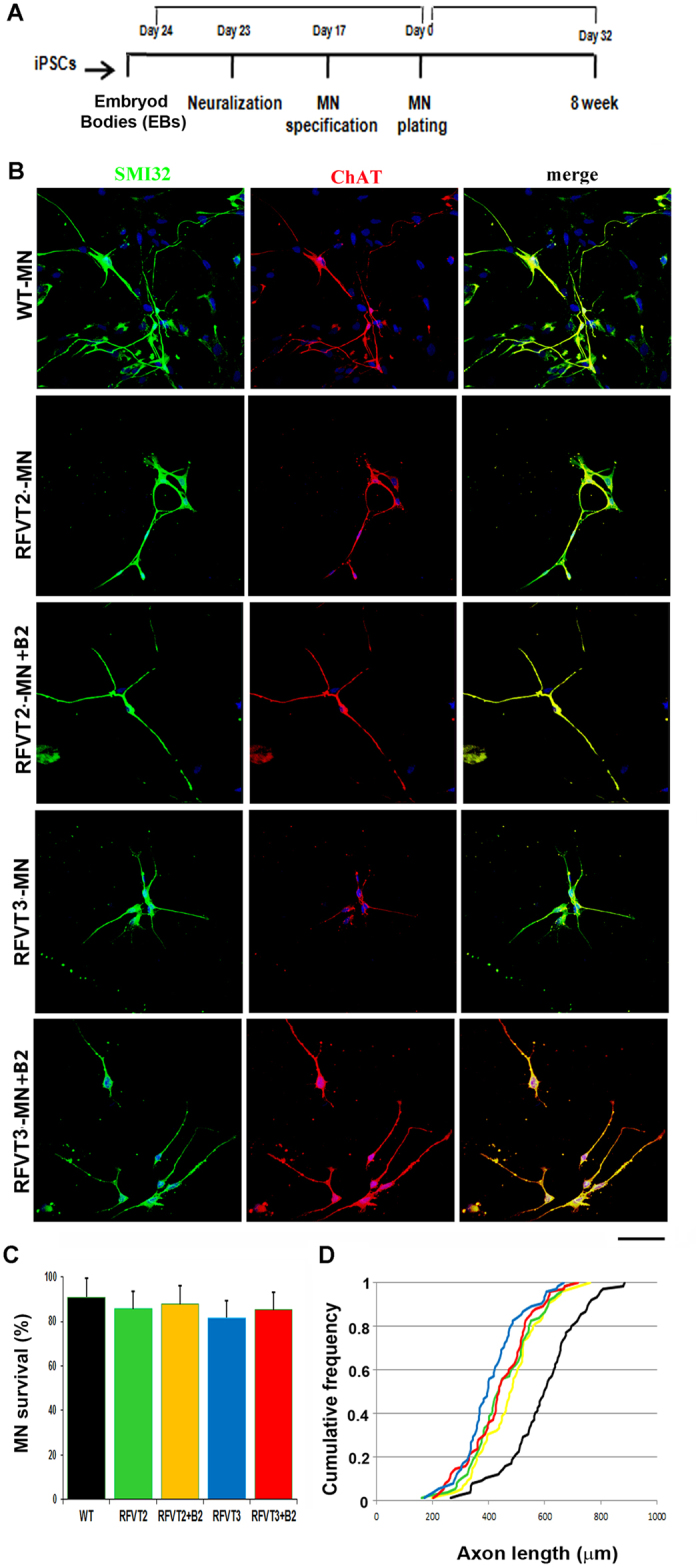
Characterization of BVVL iPSC-derived motor neurons (MNs). (**A**) Experimental outline for WT and BVVL-MN differentiation and characterization. (**B**) Immunocytochemistry of WT-MNs and BVVL-MNs (RFVT2-MNs, RFVT3-MNs). The cells were positive for typical MN markers: SMI32 (green) and ChAT (red) (merge, yellow signal). Nuclei are labeled with DAPI (blue). The analyses were performed in basal condition and after supplementation with riboflavin (B2). Scale bar: 75 μm. (**C**,**D**) Morphometric analysis of WT-MNs and BVVL-MNs. (**C**) Eight weeks after differentiation, BVVL-MN survival was not significantly reduced compared with WT-MNs (Student t test). Values represent means ± SD from five independent experiments performed in triplicate. (**D**) After 8 weeks of culture, BVVL-MNs exhibited a significantly reduced axon elongation with respect to WT cells. The improvement of axon elongation was observed after riboflavin supplementation, in particular in the patient with mutant RFVT3. Kolmogorov–Smirnov test. Five independent experiments performed in triplicate.

We reprogrammed primary fibroblasts from two BVVL patients (carrying the c.796C/c.9555C > T mutations in *RFVT2* and the c.1555C > T/c.1255G > A mutations in the *RFVT3* gene, respectively^11^; to generate iPSC lines, using a protocol based on the transfection of episomal reprogramming vectors^20^. Two WT iPSC lines reprogrammed from neonatal foreskin fibroblasts and unaffected adult skin fibroblasts were used as controls (Supplementary Table 1). After being reprogrammed, the colonies were selected for further expansion. At least three clones were isolated for each patient. The iPSCs could differentiate into cell types from all three embryonic germ layers, confirming their *in vitro* pluripotency. The cells expressed pluripotency markers such as NANOG and SSEA-3, as demonstrated by immunocytochemistry analysis (Fig. 1S).

Because MNs are selectively affected in riboflavin deficiency pathogenesis, we evaluated whether our BVVL iPSCs could be lineage-committed toward the MN fate in order to define the effect of *RFVT* mutations in this cell model. BVVL and WT iPSCs were differentiated into spinal MNs (BVVL-MNs and WT-MNs, respectively) with a multistage differentiation protocol previously developed for human pluripotent stem cells^20^ (Fig. 1A).

After 5 weeks under differentiation conditions, MNs were developed that expressed the pan-neuronal marker TUJ1 and the MN-specific transcription factor ISL1. Most of these TUJ1/ISLET1-positive neurons expressed choline acetyltransferase (ChAT) and were positive for the MN marker SMI32, indicating MN maturity (Fig. 1B). To evaluate the phenotype of BVVL, we included both basal and riboflavin-supplemented conditions (Fig. 1B).

The *in vitro* differentiation protocol generated a high proportion of MNs but also a mixed cell population containing non-MN cells. To further select MNs, we employed a physical strategy based on gradient centrifugation. This selection step allowed the enrichment of cells that were double-positive for ChAT and SMI32 (WT: 89.4 ± 6.3%; BVVL: 88.2 ± 8.6%). Less than 1% of iPSC-derived cells were positive for the astrocyte marker glial fibrillary acidic protein.

To reveal disease phenotypes associated with BVVL-MNs, we measured basal MN survival by counting SMI32-positive and ChAT-positive cells. No significant survival reduction was detected in either culture condition (Fig. 1C).

To investigate whether RFVT mutations impair axon stability and elongation, we performed morphological analyses. After 8 weeks of culture, BVVL-MNs exhibited a significantly reduced axon elongation that was improved but partially rescued by riboflavin supplementation, in particular in the patient with mutant RFVT3 (Fig. 1D). Overall, BVVL-MNs exhibited no major defects in cell survival but did show a significant axon elongation defect that riboflavin treatment ameliorated.

### Dysregulated gene expression profiles in BVVL-MNs

To identify molecular pathways that are potentially relevant for BVVL pathogenesis, we applied massive parallel RNA sequencing to MNs derived from BVVL patients and controls in the basal condition and after riboflavin treatment.

**Figure 2:**
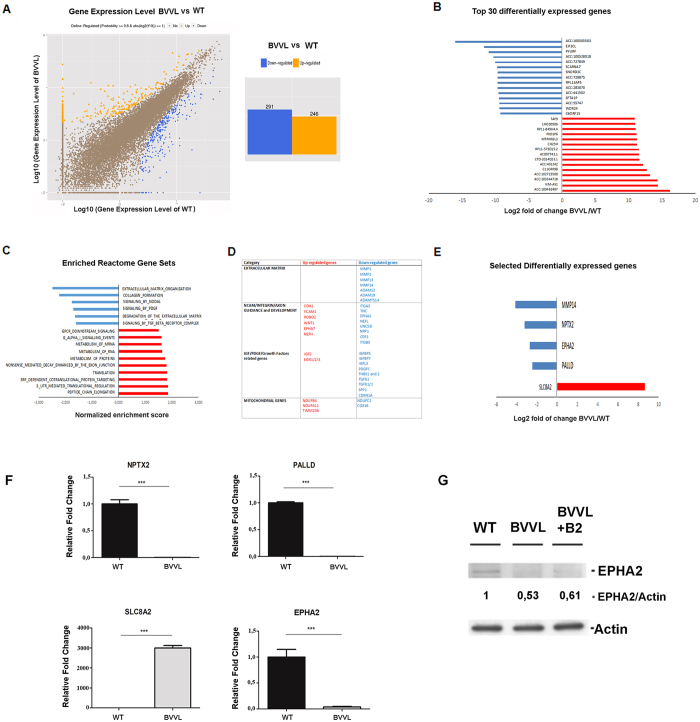
Transcriptional analysis of BVVL-MNs vs WT-MNs by RNA-seq analysis shows dysregulation of specific mRNA subsets. (**A**) *Left*: Graphical representation of the transcriptomic analysis of differentially expressed genes among BVVL-MNs vs WT-MNs. *Right*: A total of 537 differentially expressed genes (fold change ≥2 and diverge probability ≥0.8) were observed in BVVL-MNs compared to WT-MNs; 246 were up-regulated, and 291 were down-regulated in BVVL-MNs. (**B**) Top 30 genes (based on fold change) misregulated in BVVL-MNs vs WT-MNs with an FDR 5%. (**C**) Selected gene sets obtained using the GSEA algorithm. Gene sets that were enriched in BVVL-MNs include terms related to protein and RNA metabolism, while depleted genes were associated with the extracellular matrix and growth factors. (**D**) Reactome gene categories and selected genes dysregulated in BVVL-MNs. (**E**,**F**) Comparison between RNA-seq (**E**) and quantitative RT-PCR (**F**) data for a select number of transcripts involved in neurite outgrowth and axon guidance in BVVL-MNs with respect to WT-MNs. Error bars represent SEM. ***P < 0.001; **P < 0.001, *P < 0.05. (**G**) Western blot analysis of EPHA2 in BVVL and WT-MNs showing a reduced protein level in diseased cells, without significant modifications after riboflavin treatment. Numbers indicate protein levels normalized to actin.

RNA sequencing disclosed 537 differentially expressed transcripts (fold change ≥2 and diverge probability ≥0.8), 246 of which were up-regulated and 291 down-regulated in BVVL cells (Fig. 2A, Supplementary Table 2). As a measure of the transcripts most affected by RFVT mutation, we defined the identity of the 30 transcripts most increased and decreased in abundance at a false discovery rate (FDR) of 5% (Fig. 2B). To investigate the RNA-seq data for biological meaning, we used two bioinformatics tools that query for enriched gene ontology terms: gene-annotation enrichment analysis with DAVID^21^ and Gene Set Enrichment Analysis (GSEA)^22^.

Transcripts implicated in ‘cytoskeleton organization’, ‘transcriptional regulation’, and ‘protein translation’ as well as genes implicated in ‘mitochondrial function’ were the most dysregulated terms.

Taking advantage of GSEA, we identified gene sets that were significantly altered in BVVL-MNs (normalized enrichment score <1.5; Fig. 2C). Gene sets that were enriched in BVVL-MNs included terms related to “protein and RNA metabolism” while depleted genes were associated with “extracellular matrix and growth factors” (Fig. 2C,D). Several transcripts encoding for matrix metalloproteinases and metalloproteases of the ADAMS family, involved in tissue remodeling and dysregulated during neurodegeneration and in MN disease^23^, were specially downregulated in BVVL-MNs (Fig. 2D), supporting their also taking part in BVVL disease. Dysregulation of several growth factor transcripts related to IGF1 and TGF-β pathways was also observed in our *in vitro* model. One of the most represented gene sets is involved in neurite outgrowth and axon guidance and includes alteration of neurofilament (NF) mRNA, in particular in the NF heavy subunit (NFH), relevant constituents of the cytoskeletal structure, and molecules of the ephrin (EPHA) pathway that are important for axon guidance. Because NF imbalance has been associated with MN diseases^24^,^25^, we analyzed these events in-depth, as described below. Expression differences were validated by quantitative RT-PCR (Fig. 2E,F), confirming the downregulation of neuronal pentraxins (*NPTX2*), Palladin (*PALLD*), and Ephrin-A2 (*EPHA2*), as well as the up-regulation of SLC8A2 in BVVL-MNs in comparison with WT cells. Interestingly, all of these genes are related to neurite outgrowth and guidance. Given the potential relevance of EPHA2 in BVVL axonal defects, we also evaluated the protein level of EPHA2, confirming its downregulation in BVVL cells, without significant modifications after riboflavin treatment (Fig. 2G). Overall, our gene expression profile data indicate the presence of significant alterations in cytoskeletal organization and axonogenesis in the BVVL-MNs, suggesting that these could be key events in their degeneration.

### Riboflavin treatment rescued a specific subset of genes related to axonal growth and metabolism

**Figure 3:**
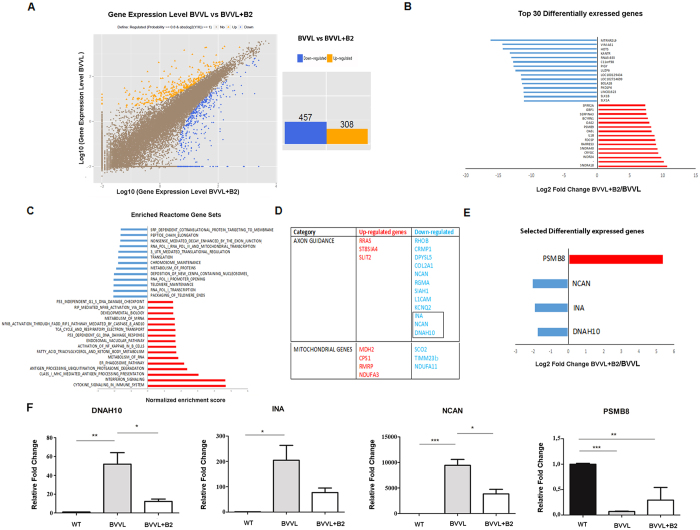
Transcriptional analysis of BVVL-MNs before and after riboflavin treatment unravels the rescue of genes related to axonal growth and metabolism. (**A**) *Left*: Graphical representation of the transcriptomic analysis of differentially expressed genes among BVVL-MNs before and after riboflavin (B2) treatment. *Right*: A total of 765 differentially expressed genes (fold change ≥2 and diverge probability ≥0.8) were observed in BVVL-MNs compared to B2-treated MNs (BVVL+B2). (**B**) Top 30 genes (based on fold change) differentially expressed in BVVL-MNs vs BVVL-MNs+B2 with an FDR 5%. (**C**) Selected gene sets obtained using the GSEA algorithm. Gene sets that were enriched in BVVL-MNs+B2 include terms related to protein, RNA, and mitochondrial metabolism, while depleted genes were associated with axon guidance. (**D**) Reactome gene categories and selected genes differentially expressed in BVVL-MNs include genes related to axon metabolism and growth as well as to mitochondrial function. (**E**,**F**) Comparison between RNA-seq (**E**) and quantitative RT-PCR (**F**) data for a select number of transcripts involved in neurite outgrowth and axon guidance in BVVL-MNs before and after treatment with B2 (BVVL-MNs+B2), in comparison with WT-MNs. B2 treatment improved the gene expression profile of DNAH10, INA, NCAN, and PSMB8, which became more similar to WT cells. Error bars represent SEM. ***P < 0.001; **P < 0.001, *P < 0.05.

The comparison of treated versus untreated BVVL-iPSC MNs (BVVL-MNs B2 vs BVVL-MNs) revealed 765 differentially expressed transcripts (Fig. 3A, Supplementary Table 3). Remarkably, the genes that were most up-regulated in treated cells include some that were down-regulated in the comparison between BVVL-MNs and WT-MNs, suggesting rescue with a shift toward the WT gene expression profile. The direct comparison between the treated BVVL-MN and WT-MNs confirmed that riboflavin supplementation shifted the expression profile toward WT (Fig. S2).

To determine the most significant effects of riboflavin treatment in terms of transcriptional changes, we identified the top 30 transcripts most increased and decreased in abundance at a FDR of 5% (Fig. 3B). GSEA identified gene sets that were significantly modified in BVVL-MNs after riboflavin treatment (normalized enrichment score <1.5; Fig. 3C).

Gene sets that were modified after treatment in BVVL-MNs included terms related to protein and RNA metabolism, mitochondrial function, and autophagy (Fig. 3C). In particular, the reactome gene category that was significantly modified was related to axon guidance and mitochondrial metabolism (Fig. 3D).

Quantitative RT-PCR was used to validate the expression of some differentially expressed genes related to axon guidance, neuronal metabolism (DNAH10, INA, NCAN), and proteasome cell function (PSMB8), confirming that their expression pattern shifted toward the normal WT profile after riboflavin treatment (Fig. 3E,F). Overall, these data suggest that the therapeutic action of B2 supplementation also is exerted through the rescue of genes that govern axon growth and maintenance.

### Alteration of neurofilament expression pattern in BVVL-MNs and its rescue after riboflavin treatment

RNA-seq analysis showed that a significant proportion of all transcripts involved in cytoskeletal and axon guidance were altered in BVVL-MNs (Fig. 2C–G). Perturbation of NF balance in *in vitro* and *in vivo* models results in phenotypes closely resembling human MN pathologies^25^,^26^ and has previously been suggested as a component of human ALS disease^24^,^25^.

**Figure 4:**
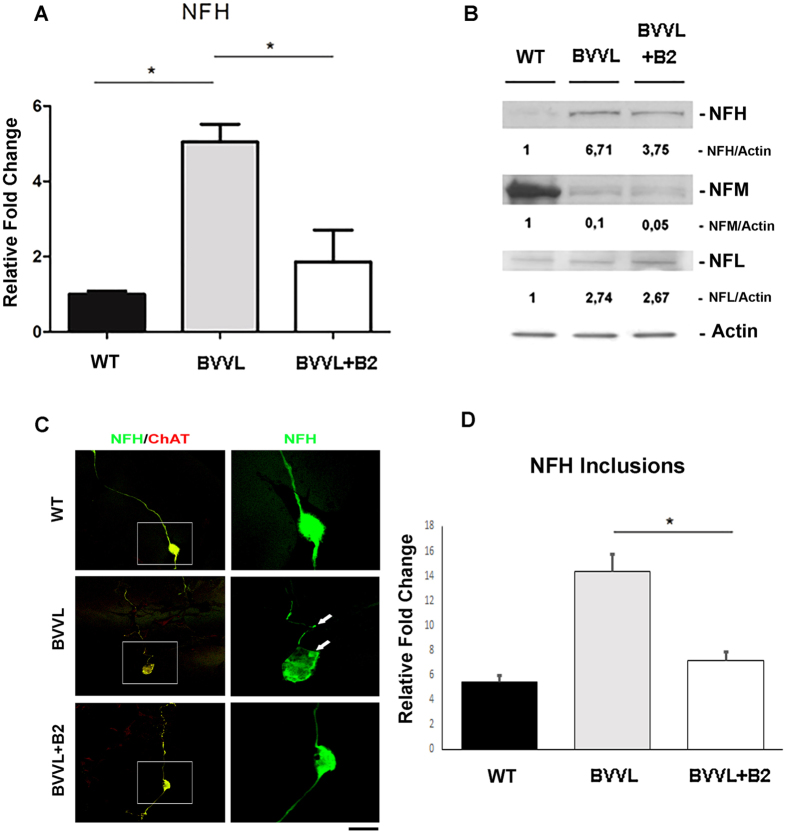
Neurofilament expression pattern is altered in BVVL MNs and is rescued by riboflavin treatment. (**A**) Real-time RT-PCR showed the up-regulation of NFH mRNA in BVVL-MNs vs WT-MNs. This upregulation was significantly reduced in BVVL-MNs+B2. Error bars represent SEM. *P < 0.05. (**B**) Western blot analysis of NFH (200), NFM (medium) (168), and NFL (light) (68) in BVVL-MNs with respect to WT-MNs and BVVL-MNs+B2. Numbers indicate protein levels normalized to actin. NFH was upregulated in BVVL-MNs relative to WT-MNs. This up-regulation was reduced after B2 treatment. The expression level of NEFM and NEFL was imbalanced respect to NFH. (**C**) Immunofluorescent images of NFH (green) and ChAT+ (red) in WT-MNs, BVVL-MNs and BVVL-MNs+B2. NFH inclusions (green) were observed in the cell body and in neurites of BVVL-MNs. This feature was ameliorated by B2 treatment. (D) Quantification of NFH inclusion–containing in cell bodies and neuritis in WT-MNs, BVVL-MNs and BVVL-MNs+B2. Error bars represent SEM. *P < 0.05. Scale bar: 75 μm.

Our RNA-seq data showed that the NFH was specifically up-regulated in our BVVL-MNs (Fig. 2D), as confirmed by real-time RT-PCR (Fig. 4A) and western blot analysis (Fig. 4B). In particular, the expression level of NF medium (NFM) and NF light (NFL) subunits was imbalanced with respect to NFH (Fig. 4B), suggesting that the altered proportion of NF subunits might have pathogenetic relevance, as already demonstrated in ALS^25^.

Furthermore, the NF immunofluorescent signal in MNs revealed that the expression levels of NFH were altered (Fig. 4C,D). In particular, progressively increased numbers of NFH inclusions in both cell bodies and neurites of BVVL-MNs was observed. Riboflavin treatment mitigated NFH aggregation and neurite degeneration (Fig. 4C,D). These results highlight that NF misregulation is a critical component of MN degeneration in BVVL.

### Dysregulation of mitochondrial dynamics and autophagy in BVVL-MNs and improvement after riboflavin supplementation

To define whether the transcriptional changes in mitochondrial genes identified by RNA-seq in BVVL-MNs (Figs 2 and 3) were indicative of mitochondria disturbances, we analyzed whether a perturbation in mitochondrial dynamic and function was present at proteomic and biochemical level. Riboflavin is the precursor of the active coenzymes FMN and FAD, which are essential cofactors for the electron transport chain and the lipid metabolism^2^. Moreover, patients with BVVL syndrome present biochemical defects that mimic MADD syndrome, an inherited disease of mitochondrial fatty acid beta-oxidation that responds to riboflavin supplementation^10^. Thus, we determined whether a similar perturbation^7^ was detectable also in BVVL-MNs.

**Figure 5:**
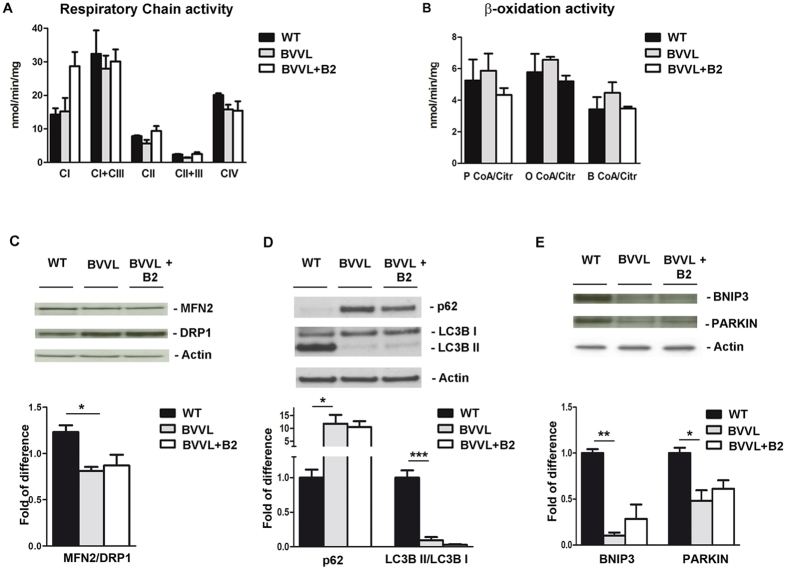
Mitochondrial function and autophagy in BBVL MNs before and after B2 treatment. Respiratory chain activity (**A**) and β-oxidation activity (**B**) analysis in BVVL-MNs, BVVL-MNs+B2, and WT-MNs. No defect in respiratory chain and β-oxidation activity was observed in BVVL cells. The enzymatic activity of respiratory complexes I–IV (cI–IV) and acetyl CoA carboxylases (P CoA, P CoA, and B CoA) was determined and normalized to citrate synthase activity. Values are expressed as nmoles/min/mg protein. (**C**) Western blot analysis of MFN2 and DRP1 in BVVL-MNs, BVVL-MNs+B2, and WT-MNs. The MFN2/DRP1 ratio was reduced in BVVL-MNs relative to WT-MNs (*P < 0.05, ANOVA). (**D**) LC3BII/I and p62 expression in WT-MNs, BVVL2-MNs, and BVVL2-MNs+B2. The LC3B-II/I ratio was reduced whereas p62 expression was increased in BVVL-MNs with respect to WT (***P < 0.0001, *P < *0.05*, ANOVA). After riboflavin treatment, LC3B-II/I ratio and p62 expression showed a shift towards a WT pattern. (**E**) Western blot analysis of Bnip3 and Parkin in WT-MNs, BVVL-MNs, and BVVL-MNs+B2 (**P < 0.001, *P < *0.05*, ANOVA). The levels of both proteins were reduced in BVVL-MNs with respect to WT-MNs.

First, we assessed for the presence of an OXPHOS or fatty acid β-oxidation impairment in BVVL-MNs, as observed in MADD. Mitochondrial respiration and β-oxidation were not different in BVVL-MNs (with or without riboflavin treatment) with respect to control cells (Fig. 5A,B).

To then analyze whether a dysregulation of the fusion/fission mitochondrial events takes place in the BVVL-MNs, as occurs in MADD, we examined the expression level of mitofusin2 (MFN2), one of the key regulators of mitochondria fusion degraded during inhibition of fusion^27^, and dynamin-related protein 1 (DRP1), which mediates outer mitochondrial membrane fission (Fig. 5C). Our results showed that the MFN2/DRP1 ratio was decreased in BVVL-MNs when compared with control cells but that this pattern was not changed by riboflavin treatment (Fig. 5C). These findings are similar to what has been observed in MADD cells^7^.

We afterward investigated whether the fusion inhibition events in mitochondria correlate with changes in autophagy. To check for the induction of autophagy, we compared the expression of the autophagy markers LC3B and p62/SQSTM in BVVL-MNs and WT-MNs. A reduction in the LC3B-II/LC3B-I ratio and increase in autophagic substrate p62 demonstrated reduced autophagy in BVVL-MNs with respect to WT-MNs (Fig. 5D). After riboflavin treatment, the LC3B-II/LC3B-I ratio and p62 expression in BVVL-MNs shifted towards a WT pattern (Fig. 5D). Mitochondrial clearance by autophagy is mediated by the induction of proteins that accumulate on mitochondria before their removal^28^. We observed that protein levels of PARKIN and BNIP3 were negatively regulated in BVVL-MNs (Fig. 5E).

Taken together, our data suggest that a reduction of fusion and mitophagy was present in BVVL-MNs, supporting their limited capacity for renewing mitochondria. Riboflavin exposure normalized the mitophagy rate. These findings showed that pathogenetic pathways in BVVL involve mitochondria turnover but not their functionality. Overall, mitochondrial turnover could represent key pathogenetic components and potential therapeutic targets of BVVL.

## Discussion

BVVL and FL are rare genetic MN diseases caused by mutations in *RFVT2*/*3* genes; however, the mechanisms by which mutant RFVT proteins induce MN degeneration are largely unknown^1^. Riboflavin (B2) supplementation seems to ameliorate the clinical phenotype even if the exact mechanisms of action on MNs have not yet clarified^1^.

Patient-specific iPSCs represents a novel tool for *in vitro* disease modelling and therapeutic discovery in neurodegenerative diseases that involve a specific neuronal population^29^. As an innovative platform to gain insights into the pathogenetic mechanisms underlying BVVL, in this study, we generated for the first time an *in vitro* BVVL disease model using human patient–specific iPSC-derived MNs. Because surface specific MN markers have not yet been established, we isolated MNs with gradient centrifugation, confirming the generation of pure MNs by immunocytochemical and molecular marker expression.

This system allowed analysis of the impact of RFVT2/3 variants on MN phenotype and to study the effects of riboflavin supplementation. Our studies demonstrated that the BVVL-MNs showed a significant reduction in their axonal length, improved by riboflavin treatment. This phenotype is similar to that observed in MN disease such as SMA^20^,^30^,^31^, and this neuropathological feature could be responsible for BVVL neurological motor signs. In fact, several studies have demonstrated a reduction in axonal growth in the MNs derived from SMA-iPSCs relative to control iPSCs^20^,^32^.

Considering the cytoskeletal-dependent neuronal pathology observed in BVVL in a broader context, we can group BVVL with those pathologies in which cytoskeletal affect neuropathological features: these defects could be responsible for the neurological motor signs of BVVL. In general, the cytoskeleton is important in a broad series of events regulating neurogenesis and maintenance of neuronal function; therefore, alterations in genes controlling cytoskeletal dynamics can easily lead to severe neurologic diseases^33^, particularly in combination with defects in the developing nervous system. Microtubules dysfunction has been observed in a model of Parkinson’s disease (PD) in which inhibition of the mitochondrial Complex I leads to accumulation of reactive oxygen species^34^,^35^. Moreover, a recent study using progressive motor neuronopathy mice carrying a missense loss-of-function mutation in tubulin binding cofactor E (TBCE) highlighted the potential contribution of cytoskeletal defects to sensory neuropathy in human MN disease^36^,^37^. The cytoskeletal alterations observed in many neurological disorders may be primary or secondary factors in neurological motor signs, but regardless, identifying therapies that can correct these microtubule abnormalities could improve the clinical manifestations. We hope that future research will target specific proteins or pathways affecting the cytoskeleton and ways to shift the status of the altered cytoskeleton back toward normal, thus leading to effective therapies for a wide spectrum of neurodegenerative diseases.

The improvement in RNA sequencing technologies has allowed identification of specific transcriptional changes in human MN diseases such ALS and SMA, contributing to defining possible disease hallmarks and pathways associated with differential MN vulnerability, thus presenting a number of striking targets for future therapeutic strategy^38^,^39^. In particular, Eggan and colleagues observed a specific transcriptional signature indicative of increased oxidative stress, reduced mitochondrial function, altered subcellular transport, and activation of the ER stress in ALS-MNs^39^. Similar changes in hyper-activation of the ER stress pathway have been described in SMA-MNs^38^ in association with RNA splicing abnormalities^20^ leading to the hypothesis that they could be mechanisms of selective MN death.

In this study, for the first time, by applying RNA sequencing analysis to our model in the basal condition and after riboflavin treatment, we identified specific BVVL gene expression changes in RNA/protein metabolism, axonal and cytoskeletal structure, and mitochondrial/oxidative function. Novel candidate genes, with significantly altered expression in BVVL-MNs compared to WT-MNs were selected. In particular, we detected the significant downregulation of neuronal pentraxin 2 (*NPTX2*), palladin (*PALLD*), and EPH receptor A2 (*EPHA2*), all genes related to neurite outgrowth and axon guidance. NPTX and their corresponding receptors have been studied as synapse-associated proteins in the nervous system and are relevant in promoting neuronal growth and circuitry organization. NPTX2 downregulation can negatively affect these events^40^. PALLD is a critical component of the developing nervous system, with an important role in axonal extension^41^. A reduction in the expression of PALLD, as observed in our BVVL-MNs, can result in a failure of neurite outgrowth. The significantly altered expression of the axon guidance protein EPHA2, observed in our model, could have an important scientific relevance based on its critical function during MN axon outgrowth to the hindlimb with potential roles in pathfinding^42^. Indeed, the perturbation of axon guidance protein, which guide growing axons during development and control the structural plasticity of synaptic connections in adults, has been implicated in several neurological diseases^43^ such as autism spectrum disorders and epilepsy^44^ and MN disorders, in particular ALS^45^, even if the molecular mechanisms are poorly understood. For several neurological diseases, different studies have identified causative mutations in genes for axon guidance, but for other disorders such as BVVL, additional studies are needed to demonstrate the role of these proteins in the pathogenetic mechanisms and to identify new targets for therapeutic intervention.

The comparison of B2-treated versus untreated BVVL-MNs allowed us to identify differentially expressed transcripts. Interestingly, the genes that were most up-regulated in treated cells include some that were down-regulated in the comparison between BVVL-MNs and WT-MNs, supporting a rescue of gene expression profile with a shift toward the WT pattern. Among the transcripts that were significantly modified towards a WT pattern, we identified DNAH10 (Dynein axonemal heavy chain 10) and INA (alpha-internexin), as well as Neurocan (NCAN), a proteoglycan that modulates axonal outgrowth. DNAH10 is a microtubule-associated protein and key component of MN axons^46^. Interestingly, mutations in DNAH10 have been recently associated with motor-sensory neuropathies (Charcot–Marie–Tooth disease)^46^. Alpha-internexin (INA) is a neuronal intermediate filament protein that plays a similar role to light NF^47^. Its expression precedes that of NF light during neuronal development^47^. NCAN is an important nervous tissue–specific chondroitin sulfate proteoglycan that modulates axonal outgrowth^48^. In general, these findings support the hypothesis that the therapeutic action of B2 supplementation is elicited mainly through the normalization of the expression of key genes that control axonal elongation and function.

Furthermore, our data showed an alteration in the delicate NF stoichiometric balance, a key pathogenetic event that has been previously suggested to be central in several MN diseases including ALS^24^,^25^. NF aggregation has been detected in ALS patients and transgenic animals^49^,^50^,^51^, and transgenic alteration of NFs in neurons, especially overexpression, can result in ALS^24^,^52^,^53^,^54^. These data supported the idea that the altered stoichiometry of NF subunits generated by variation in NF subunit expression results in their pathological aggregation^25^,^55^,^56^,^57^,^58^. Indeed, ALS spinal MNs displayed NF aggregation followed by neurite degeneration in association with a decreased stability of NFL mRNA and altered protein proportion of NF subunits. Of importance, conditional expression of NFL in ALS MNs corrects the NF subunit proportion, mitigating NF aggregation and neurite degeneration^25^.

In agreement with this observation in ALS-MN, our BVVL-MNs presented an increased level of NFH with aggregation that is likely responsible for neurite degeneration. The substantially higher NF content in MNs demands tight regulation; thus, its deregulation can account for the selective MN degeneration present in BVVL. This finding confirms the potential of targeting NF regulation for therapeutic intervention in MN diseases, but also in BVVL.

As noted, riboflavin is the precursor of the active coenzymes FMN and FAD that are essential cofactors for the electron transport chain and the lipid metabolism^2^, leading us to investigate these pathways. We identified perturbation in the expression of genes related to mitochondrial pathways as well as in the mitochondrial dynamic demonstrated by RNA sequencing and western blot studies, while their functionality in terms of respiratory chain and β-oxidation activity was not altered. Our proteomic data suggest the presence of an altered mitochondria turnover characterized by inhibition of fusion events leading to damage of the mitochondria and block of autophagy and mitophagy in BVVL-MNs, resulting in a reduced capability of removing dysfunctional mitochondria. The wellness of the mitochondrial network represents a requisite for neuronal health and survival. A timely elimination of aged and dysfunctional mitochondria through mitophagy, a selective and regulated form of autophagy, is an essential protective mechanism even more relevant in non-mitotic cells such as neurons, in which damaged cellular components cannot be diluted through recurring cell division cycles^59^,^60^. In fact, accumulation of large defective mitochondria has been associated with cell senescence/ageing and with PD, in fact brain belonging to PD patients present reduced expression of autophagy protein^61^,^62^. Some forms of MN disorders are characterized by autophagy impairment, which reduces their capacity to maintain protein homeostasis, and results in eventual neuronal cell loss^63^,^64^, although a causal relationship between autophagic dysfunction and disease has not been fully established. As showed in other MN disease, our data indicated that one possible pathogenetic mechanism in BVVL-MN degeneration is their incapacity to effectively cope with increased damage to the mitochondria, leading to cell apoptosis/necrosis.

In conclusion, iPSC patient-derived MNs allowed us to unravel novel molecular and cellular pathogenetic events that represent possible targets for therapeutic intervention, in addition to riboflavin supplementation for BVVL and other MN diseases. The strategy of using iPSCs obtained from patients to differentiate MNs offers the possibility to study the cell type of interest and to dissect the cellular (morphological) and molecular properties of the affected cells. Moreover, this approach allowed us to perform detailed analyses following riboflavin supplementation. Despite these advantages, limitations in understanding the pathogenesis of MN diseases remain. We analyzed the MN cultures in the commonly used 2D *in vitro* context, but the physiological environment obviously is 3D and characterized by a tight communication among different cell types (i.e., neurons with glial cells). Moreover, the *in vitro* system allows direct access to the cell of interest whereas in the human body, similar access may be much more challenging. Despite these limitations of our approach, we still consider it to be a useful model for unraveling new aspects of human pathologies, especially in cases where tissue accessibility is hard to achieve. We aim to continue our investigations of BVVL pathogenesis using iPSC-derived MNs in 3D culture systems and co-culturing of glial cells and neurons.

## Materials and Methods

### Derivation of human fibroblasts and reprogramming into iPSCs

The studies involving human samples were conducted in compliance with the Code of Ethics of the World Medical Association (Declaration of Helsinki) and with national legislation and institutional guidelines. Human fibroblast cell lines were obtained from Eurobiobank with informed consent (ethical committee approved at the IRCCS Foundation Ca’ Granda Ospedale Maggiore Policlinico). Fibroblasts were generated from dermal biopsies following informed consent as described previously^65^. Generation of iPSCs was performed by non-viral transduction with six reprogramming factors (OCT4, SOX2, NANOG, LIN28, c-Myc, and KLF4)^66^. iPSC colonies with embryonic stem cell–like morphology were expanded and successively detached for the analysis. iPSCs were grown on Matrigel (BD Biosciences) with E8 media (Invitrogen). All cell cultures were maintained at 37 °C, 5% CO_2_.

### MN differentiation

MN differentiation was performed as previously described^65^,^66^. Briefly, cells were plated with neuronal medium DMEM-F12 (Life Technologies) supplemented with MEM nonessential amino acids solution (Life Technologies), N2 (Life Technologies), and 2 mg/ml heparin (Sigma-Aldrich). After 10 days, 0.1 μM retinoic acid (RA; Sigma-Aldrich) was added for neural caudalization. On day 17, posteriorized neuroectodermal cells were collected and suspended for a week in the same medium with 0.1 μM RA and 100–200 ng/ml Sonic hedgehog (R&D Systems), followed by the addition of 10 ng/ml brain-derived neurotrophic factor (PeproTech) and 10 ng/ml glial derived neurotrophic factor (PeproTech) on day 24. A centrifugation gradient was employed to maximize MN enrichment^20^,^67^,^68^.

The BBVL cells were cultured at two riboflavin concentrations: 5 nmol/l (identified as the basal condition) and 1 μmol/l (identified as the therapeutic condition). The high concentration of riboflavin (1 μmol/l) was chosen because this concentration is above the level of riboflavin observed in plasma from individuals treated with riboflavin^7^,^69^. Riboflavin 1 μmol/l is a standard concentration present in culture media. The low concentration of riboflavin (5 nmol/l) was chosen because it represents a level close to that for riboflavin observed in plasma in BVVL patients^7^,^10^.

### Immunocytochemistry and phenotypic analysis

#### Immunocytochemistry of iPSCs and MNs

Cells were fixed in 4% paraformaldehyde for 10 min, permeabilized with Triton 0.25%, and then blocked with 10% bovine serum albumin in 1× phosphate-buffered saline and 0.3% Triton X-100 for 1 h at room temperature. We incubated the cells with primary antibodies to NANOG (1:100, Abcam), SSEA-3 (1.100, Covance), ChAT (1:200, Chemicon & Millipore), SMI32 (Covance, 1:500), and Neurofilament-200 (1:1000 Sigma) overnight, and then with anti-rabbit or anti-mouse or -goat Alexa Fluor 488 or 594 (1:400; Life Technologies) secondary antibody for 1.5 h at room temperature.

#### Morphometric analysis

Phenotypic analysis was performed as previously described^65^,^66^,^70^. We quantified MNs by determining cells positive for MN marker and counting 10 randomly selected fields/well (3 wells/condition/experiment in four to five experiments). The morphometric axon length was determined using soma diameter and the distance between two points. For all imaging, we used a confocal LEICA SP2 and Nikon microscope.

### mRNA-seq library preparation, sequencing, and gene expression analysis

Total RNA was isolated from pooled cells using the PicoPure RNA Isolation Kit (Arcturus), which allows the recovery of RNA from a small amount of cells, and quantified using Bioanalyzer (Agilent). A TruSeq RNA sample preparation Kit v2 (Illumina) was used to obtain cDNA libraries from total RNA, and the Nextera DNA sample preparation kit (Illumina) was employed to prepare the sequencing library. Libraries were sequenced on an Illumina HiSeq 2000 employing the standard Illumina pipeline software on an Illumina platform (performed at BGI). Two biological replicates for patients and controls, each comprising four pooled samples, were analyzed. Each replicate was analyzed separately and then pooled. Raw data were processed using TopHat version 2.0.9 and Cufflinks to align genome reads, assemble the data into transcripts, and define expression levels. The aligned mRNA reads were used to identify differentially expressed genes.

### Bioinformatics analysis

For NGS data analysis, we used two bioinformatics tools that query for enriched gene ontology terms: gene-annotation enrichment analysis with DAVID^21^ and GSEA^22^. Enrichment for up-regulated or down-regulated sets of genes from the REACTOME pathway and GO term database was computed by running GSEA against the test statistic-ranked list of genes in the experiment. Ranking was based on the Cuffdiff 2-derived test statistic. All REACTOME and GO term gene sets with >15 members in the MSigDB package “c2.all.v3.0.symbols. gmt” and “c5.all.v4.0.symbols.gmt,” respectively, were downloaded from ftp://gseaftp.broadinstitute.org/.

### RNA isolation and quantitative real-time PCR

Total RNA was extracted from cells using the RNeasy Mini Kit (Qiagen). Concentrations were measured with a Nanodrop spectrophotometer. Only samples with ratios between 1.8 and 2.0 were further analyzed. A total of 1 μg of total RNA was reverse-transcribed using the Ready-To-Go kit (GE Healthcare). Reverse-transcribed material (5 ng for each sample) was amplified using the TaqMan Universal PCR Master Mix (Applied Biosystems) and the appropriate primers to evaluate gene expression (probe ID available upon request) in the 7500 Real Time PCR System. The expression levels for all genes were normalized to the average levels of the housekeeping gene *ACTB* and referred to the relevant control samples.

### Biochemical studies

The specific activities of individual respiratory-chain complexes and β-oxidation were evaluated in MNs, as previously described^71^,^72^. The specific activity of each complex was normalized to that of citrate synthase.

### Protein studies

Proteins extracted from MNs were separated by SDS-PAGE. The primary antibodies used in these experiments were anti EphA2 (1:1000, Abcam), anti-NFs (1:1000, Sigma), MFN2 (Sigma, 1:1000), DRP1 (Sigma, 1:1000), p62 (Millipore, 1:1000), LC3 I/II (Millipore, 1:500), PARKIN (Abcam, 1:2000), and BNIP3 (Abcam, 1:1000) using the manufacturer instructions. The following secondary antibodies were used: polyclonal anti-rabbit (1:2700, Dako) and polyclonal anti-mouse (1:3200, Dako). The nitrocellulose membrane was stripped and re-probed with anti-actin monoclonal antibody as a loading control. Densitometry was performed using ImageJ software.

### Statistical analysis

Stats Direct software was used for all statistical analyses. All counting data from immunocytochemical analyses and cell survival were expressed as mean ± SD or SEM. Differences between two means were analyzed using the Student’s t-test (two-tailed), and differences among more than two means were analyzed using one- or two-way ANOVA. When ANOVA revealed significant differences, pair-wise comparisons between means were performed using Tukey’s post-hoc test.

## Additional Information

**How to cite this article:** Rizzo, F. *et al*. Genome-wide RNA-seq of iPSC-derived motor neurons indicates selective cytoskeletal perturbation in Brown–Vialetto disease that is partially rescued by riboflavin. *Sci. Rep.* **7**, 46271; doi: 10.1038/srep46271 (2017).

**Publisher's note:** Springer Nature remains neutral with regard to jurisdictional claims in published maps and institutional affiliations.

## Supplementary Material

Supplementary Information
