# Supplementary material for: Genome-wide RNA-seq of iPSC-derived motor neurons indicates selective cytoskeletal perturbation in Brown–Vialetto disease that is partially rescued by riboflavin
Source: Sci Rep. 2017 Apr 6;7:46271. doi: 10.1038/srep46271 (PMC5382781; doi:10.1038/srep46271)
Supplement: Supplementary Information [file srep46271-s1.pdf]

Genome-wide RNA-seq of iPSC-derived motor neurons indicates selective cytoskeletal perturbation in Brown–Vialetto disease that is partially rescued by riboflavin

Federica Rizzo[1*], Agnese Ramirez[1]*, Claudia Compagnucci[2]*, Sabrina Salani[1], Valentina Melzi[1], Andreina Bordoni[1], Francesco Fortunato[1], Alessia Niceforo[2], Nereo Bresolin[1], Giacomo P. Comi[1], Enrico Bertini[2], Monica Nizzardo[1] and Stefania Corti[1]**

[1]Dino Ferrari Centre, Neuroscience Section, Department of Pathophysiology and Transplantation (DEPT), University of Milan, Neurology Unit, IRCCS Foundation Ca' Granda Ospedale Maggiore Policlinico, Milan, Italy.

[2]Unit of Neuromuscular and Neurodegenerative Disorders, Laboratory of Molecular Medicine, Bambino Gesu' Children's Research Hospital, Rome, Italy

*Equally contributed to the work

**Corresponding author: Neuroscience Section, Department of Pathophysiology and Transplantation (DEPT), University of Milan, Neurology Unit, IRCCS Foundation Ca' Granda Ospedale Maggiore Policlinico, Via Francesco Sforza 35, 20122 Milan Italy. Tel: +39 0255033833; Fax: +39 0255033800; Email: stefania.corti@unimi.it.

Supplementary Figure 1. Reprogramming human BVVL fibroblasts into iPSCs.

Immunocytochemical characterization of WT and BVVL-iPSCs (RFVT2 iPSC and RFVT3 iPSC). These iPSCs showed the typical pluripotent stem cell colonies morphology at the contrast phase microscope (left panels). The cells also expressed pluripotency transcription factors, including NANOG (red), and stem cell surface markers SSEA-3 (green). Nuclei were labeled with DAPI (blue). Scale bars: 100 μm.

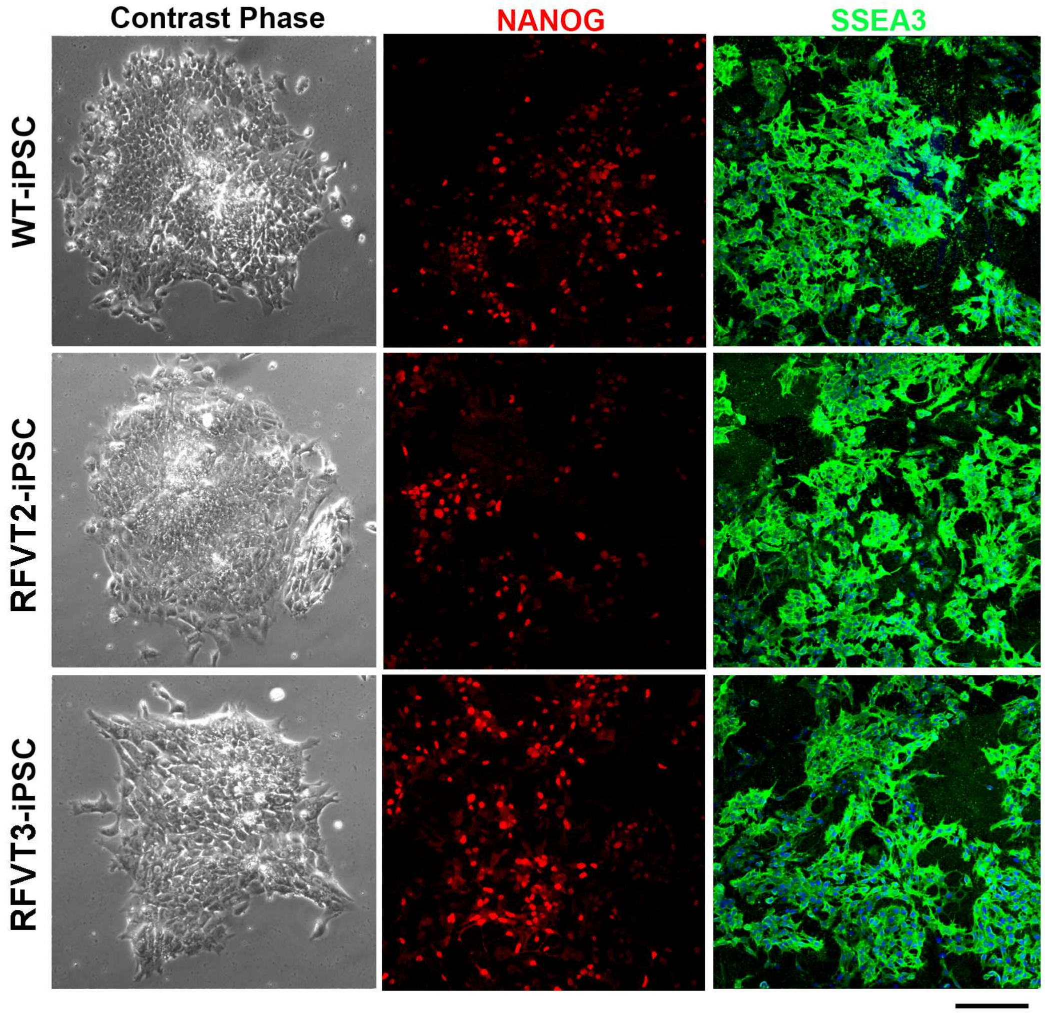

Supplementary Figure 2. Transcriptional analysis of BVVL-MNs treated with riboflavin (BVVL-MNs+B2) vs WT-MNs by RNA-Seq analysis *Left*: Graphical representation of the transcriptomic analysis of differentially expressed genes in BVVL-MNs+B2 vs WT-MNs. *Right*: 564 genes were up-regulated and 468 were down-regulated in BVVL-MNs+B2 (fold change ≥ 2 and diverge probability ≥ 0.8).

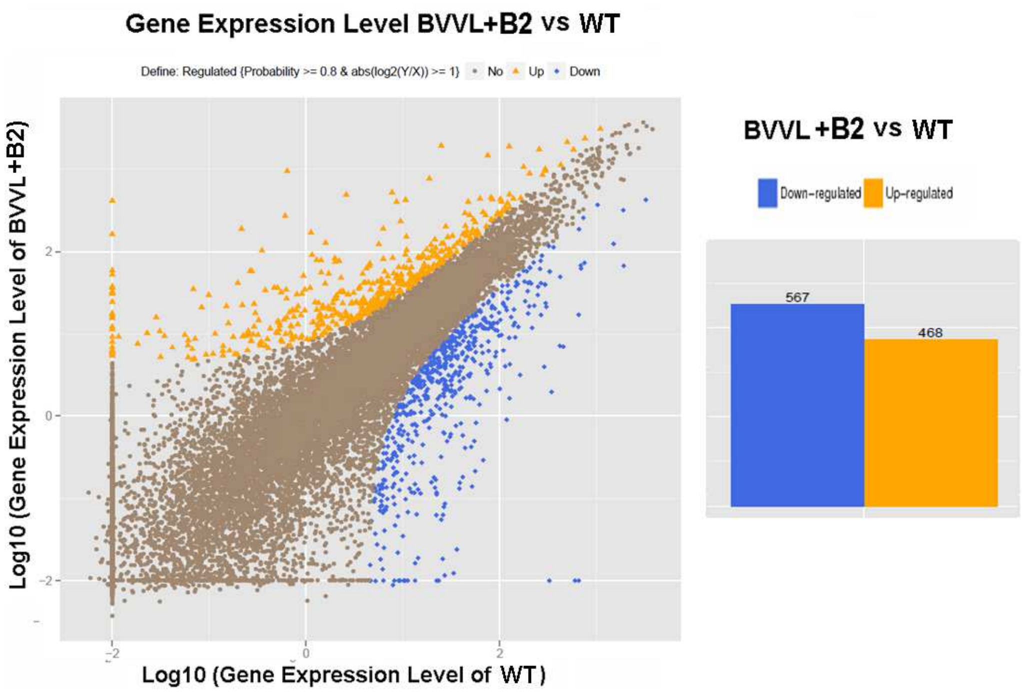

Supplementary Table 1. Characteristics of human fibroblast-derived induced pluripotent stem cells (iPSCs).

iPSC line	Diagnosis	Clinical phenotype	Mutation	Sex	Age (years)	Ref.
RFVT2 1.1	BVVL	Ciccolella 2012	c.796C>T/c.955C>T	Male	13	This report
RFVT2 1.2	BVVL	Ciccolella 2012	c.796C>T/c.955C>T	Male	13	This report
RFVT2 1.3	BVVL	Ciccolella 2012	c.796C>T/c.955C>T	Male	13	This report
RFVT3 2.1	BVVL	Ciccolella 2013	c.155C>T/c.1255G>A	Male	3	This report
RFVT3 2.2	BVVL	Ciccolella 2013	c.155C>T /c.1255G>A	Male	3	This report
RFVT3 2.3	BVVL	Ciccolella 2013	c.155C>T /c.1255G>A	Male	3	This report
19.9	Healthy Donor	-	-	Male	Newborn	Simone2014
CTRAC 1.1	Healthy Donor	-	-	Female	24	Simone 2014
CTRAC 1.2	Healthy Donor	-	-	Female	24	Simone 2014
CTRAC 1.3	Healthy Donor	-	-	Female	24	Simone 2014

Supplementary Table 2. List of genes with significantly altered expression in BVVL MNs compared to WT MNs in RNA sequencing (fold-change ≥2 and diverge probability ≥0.8)

GeneID	Gene Symbol	log2Ratio(BVVL/WT)
3956	LGALS1	-5,511881516
1277	COL1A1	-5,718496555
811	CALR	-2,086576275
5763	PTMS	-2,835204356
6678	SPARC	-3,329410144
4267	CD99	-2,856306453
7045	TGFBI	-3,753779641
2335	FN1	-2,29879703
4323	MMP14	-4,098741442
1278	COL1A2	-3,59266086
100505503	-	-15,99683934
100008589	RNA28S5	2,079455756
3488	IGFBP5	-4,230882934
378938	MALAT1	-5,389575998
1291	COL6A1	-3,167264268
6876	TAGLN	-2,476970174
51504	TRMT112	-2,065706512
3490	IGFBP7	-3,970877493
728658	RPL13AP5	-9,65577666
6206	RPS12	2,477648413
100861532	RNA45S5	-4,575717917
10631	POSTN	-2,350101669
1289	COL5A1	-3,6483772
4922	NTS	-5,174965825
4313	MMP2	-2,997023984
1292	COL6A2	-2,364963115
6281	S100A10	-2,527104303
6160	RPL31	2,891765256
1290	COL5A2	-2,123785961

6277	S100A6	-2,101616389
378706	RN7SL2	-2,49109058
8870	IER3	-2,494488882
6229	RPS24	2,974916745
11117	EMILIN1	-4,708607892
633	BGN	-5,090555267
6227	RPS21	2,472473061
7070	THY1	-2,985053366
1282	COL4A1	-3,355047938
1284	COL4A2	-2,667606172
6029	RN7SL1	-2,518951449
5460	POU5F1	2,065893094
125144	LRRC75A-AS1	2,090382734
3371	TNC	-4,128284155
5270	SERPINE2	-2,267969849
9902	MRC2	-2,806279193
7837	PXDN	-2,040156545
165	AEBP1	-2,59004056
1295	COL8A1	-7,686618279
664709	HNRNPA1P10	-6,742124342
1026	CDKN1A	-3,125632591
6382	SDC1	-3,46655732
6235	RPS29	3,680902915
5919	RARRES2	-3,541095047
6696	SPP1	-3,925652455
4666	NACA	2,991944147
284119	PTRF	-3,55690797
301	ANXA1	-2,076744601
100131205	RPL21P28	-4,23986448
6238	RRBP1	-2,355207985
4017	LOXL2	-2,266905636
6158	RPL28	2,120583275
6173	RPL36A	2,582144171

7040	TGFB1	-3,409542796
654364	NME1-NME2	-2,319593083
152007	GLIPR2	-2,495482564
822	CAPG	-2,744291944
2173	FABP7	-2,527099408
151887	CCDC80	-4,110094532
27122	DKK3	-2,858717569
10683	DLL3	-2,088703019
5355	PLP2	-2,589692127
64855	FAM129B	-2,192368691
514	ATP5E	2,546034972
2621	GAS6	-2,842197609
23022	PALLD	-2,422849513
3693	ITGB5	-2,575681424
7057	THBS1	-2,429501658
618	BCYRN1	-3,463291228
1293	COL6A3	-2,702842781
100533955	SENP3-EIF4A1	-6,728040401
143282	FGFBP3	-2,263941223
140576	S100A16	-2,817462629
1381	CRABP1	4,871077624
4747	NEFL	-2,190687053
2012	EMP1	-2,64532538
187	APLNR	-2,369856522
10867	TSPAN9	-2,402742209
8364	HIST1H4C	-6,257694326
3219	HOXB9	-2,181123444
7474	WNT5A	-3,087666315
794	CALB2	-5,444495788
23091	ZC3H13	-2,219897517
56937	PMEPA1	-5,030211468
7857	SCG2	-7,162565259
11031	RAB31	-2,388293281

27063	ANKRD1	-3,285910319
4885	NPTX2	-3,216763978
894	CCND2	-2,137449952
144983	HNRNPA1L2	-2,907569712
112597	LINC00152	-2,667731202
100529067	SERF2-C15ORF63	-6,540850999
6004	RGS16	-2,794200795
60492	CCDC90B	-2,147209829
50863	NTM	-3,270620994
26585	GREM1	-2,797120739
8459	TPST2	-2,195021231
6285	S100B	-3,189880355
30844	EHD4	-2,846707699
9547	CXCL14	-3,096502606
57333	RCN3	-3,394395781
90993	CREB3L1	-3,0650721
1969	EPHA2	-2,705517176
441502	RPS26P11	-9,516174535
4300	MLLT3	-2,375942519
1958	EGR1	2,925239546
5054	SERPINE1	-3,757067154
2791	GNG11	-2,511800979
100526842	RPL17-C18orf32	-2,512211695
8728	ADAM19	-3,742089925
79081	LBHD1	-3,697412839
26010	SPATS2L	-2,379852753
100529239	RPS10-NUDT3	-5,023028003
84870	RSPO3	-3,295400737
7044	LEFTY2	-3,757367289
4239	MFAP4	-3,086146655
857	CAV1	-2,320409163
9452	ITM2A	-3,274314784
388	RHOB	2,477603648

4071	TM4SF1	-7,061466674
9350	CER1	-5,145113378
8822	FGF17	-4,009718623
56034	PDGFC	-2,936123833
7041	TGFB1I1	-2,365801814
5654	HTRA1	-2,480776896
4907	NT5E	-7,248902671
2353	FOS	3,449433677
100532726	NDUFC2-KCTD14	-3,551913779
4837	NNMT	-5,427243076
55214	P3H2	-2,68997014
84171	LOXL4	-5,586387631
23612	PHLDA3	-3,149983195
6892	TAPBP	-2,352705691
26509	MYOF	-2,946097005
4710	NDUFB4	2,114742198
728689	EIF3CL	-11,70644237
79366	HMGN5	-3,717848181
6862	T	-2,621214414
5328	PLAU	-2,593353079
2170	FABP3	-3,181885817
9697	TRAM2	-2,476661922
2571	GAD1	-4,351704315
115908	CTHRC1	-2,712981852
25960	ADGRA2	-2,519009137
5396	PRRX1	-2,75273623
4015	LOX	-2,962410059
5936	RBM4	2,084943535
1300	COL10A1	-6,603619621
22801	ITGA11	-6,173713506
3233	HOXD4	-3,655436331
3235	HOXD9	-4,401778883
10842	PPP1R17	-2,686020327

541471	MIR4435-2HG	-2,837314305
133	ADM	-2,531802331
7763	ZFAND5	2,251589645
729739	LOC729739	-5,618787093
3037	HAS2	-2,653734697
23671	TMEFF2	-3,499693686
8829	NRP1	-2,894745275
7478	WNT8A	-2,531629917
26353	HSPB8	-4,467214466
26002	MOXD1	-4,276286102
100532735	INO80B-WBP1	-2,455256019
25907	TMEM158	-2,508368741
4053	LTBP2	-4,585706113
2162	F13A1	-5,886517454
100532737	ATP6V1G2-DDX39B	-2,419599013
100526836	BLOC1S5-TXNDC5	-3,687059565
3675	ITGA3	-3,525198273
92196	DAPL1	-6,074442301
1893	ECM1	-4,558950519
390	RND3	2,412638704
2	A2M	-4,723374035
55568	GALNT10	-2,364523547
1305	COL13A1	-3,117231941
3231	HOXD1	-3,685145712
149111	CNIH3	-3,39552372
207107	SFTA1P	-9,436665115
100528064	NEDD8-MDP1	-7,224974258
91624	NEXN	-4,277298563
3671	ISLR	-3,562588287
342979	PALM3	-2,691516764
8320	EOMES	-6,062542384
6793	STK10	-2,761027303
56849	TCEAL7	-3,356897235

100529209	RNASEK-C17orf49	-4,227345302
136319	MTPN	-3,916351994
112464	PRKCDBP	-2,984293877
653464	SRGAP2C	-2,578571932
29995	LMCD1	-2,666961791
1674	DES	-3,361762438
518	ATP5G3	2,024912991
5480	PPIC	-2,596829915
6911	TBX6	-2,544609734
3624	INHBA	-6,51542372
9235	IL32	-3,016760465
100534592	URGCP-MRPS24	-4,731569755
3398	ID2	2,780588848
9961	MVP	-2,61769399
11010	GLIPR1	-2,903370138
27344	PCSK1N	-2,75134331
64399	HHIP	-6,125196055
83468	GLT8D2	-3,053103851
81855	SFXN3	-3,566965611
126868	MAB21L3	-4,431865662
5455	POU3F3	-2,976497205
728855	LINC00623	-3,203630587
3226	HOXC10	-3,816491941
100996939	PYURF	-11,03068335
126328	NDUFA11	2,914124927
92689	FAM114A1	-2,755237258
219699	UNC5B	-2,884056503
55816	DOK5	2,222188101
100302739	PCNA-AS1	2,908668699
771	CA12	-3,383429365
3215	HOXB5	-3,72599215
9955	HS3ST3A1	-3,492316764
3237	HOXD11	-5,624181843

6423	SFRP2	2,775128732
79901	CYBRD1	-2,785234419
4826	NNAT	2,449872467
3214	HOXB4	-3,049413673
3236	HOXD10	-6,088410102
27286	SRPX2	-5,047819872
51313	FAM198B	-3,746632219
115701	ALPK2	-2,84274678
10516	FBLN5	-3,587100727
6495	SIX1	-3,959980379
1464	CSPG4	-3,856534385
93010	B3GNT7	-2,94594074
100526833	SEPT5-GP1BB	-2,915141507
83881	MIXL1	-2,820126833
2303	FOXC2	-4,028080168
10882	C1QL1	-2,995713963
304	ANXA2P2	-7,324658159
290	ANPEP	-3,011388339
171423	PDIA3P1	-3,259175223
3217	HOXB7	-3,185311874
1652	DDT	2,612930049
10417	SPON2	-4,128374301
3206	HOXA10	-2,967843217
9060	PAPSS2	-3,874900277
23209	MLC1	-4,830635452
10739	RFPL2	-5,367649596
3232	HOXD3	-3,87743896
9518	GDF15	-3,499479523
57381	RHOJ	-3,458722754
387758	FIBIN	-3,340766066
100534589	HOXA10-HOXA9	-5,943783911
26851	SNORD3B-1	-4,424966917
653659	TMEM183B	-3,705691872

7262	PHLDA2	-3,11823439
100528018	ARL2-SNX15	-10,32953343
2297	FOXD1	-3,063688006
84973	SNHG7	2,238780636
339122	RAB43	-3,070162594
8038	ADAM12	-3,503366856
1843	DUSP1	2,872306977
100885850	PTGES3L-AARSD1	-3,725721666
7058	THBS2	-3,639955283
199990	FAAP20	2,240165686
1307	COL16A1	-3,223354706
64123	ADGRL4	-5,643006131
441531	PGAM4	-7,502725094
283208	P4HA3	-7,330115936
780852	SNORD3B-2	-4,175388244
83690	CRISPLD1	2,250116379
8862	APLN	-4,888737373
2670	GFAP	-6,812863492
10874	NMU	3,325188819
727849	-	-10,03740814
4312	MMP1	-3,652080549
574036	SERTAD4-AS1	-4,442755983
441454	LOC441454	-5,433417168
131034	CPNE4	-4,150656075
3234	HOXD8	-3,744925783
8436	SDPR	-7,380871471
388135	C15orf59	-3,81490878
5696	PSMB8	-3,614435194
10637	LEFTY1	-4,604099689
642423	LOC642423	-5,22162182
8291	DYSF	-4,009038478
26289	AK5	-4,456835738
6447	SCG5	2,3842339

51393	TRPV2	-3,880365442
5069	PAPPA	-3,69492269
57214	CEMIP	-5,758704143
677767	SCARNA7	-9,757160691
10457	GPNMB	3,330504357
360132	FKBP9P1	-5,586900291
283106	CSNK2A3	-6,890530661
3589	IL11	-3,962829346
169044	COL22A1	-4,731614961
780853	SNORD3C	-9,678883364
728875	-	-9,673398063
9358	ITGBL1	-5,813893267
10653	SPINT2	2,266581616
4212	MEIS2	2,739549508
8788	DLK1	3,437757865
100874392	ANKRD20A12P	-5,062385144
283070	-	-9,642742956
389692	MAFA	-6,407421981
23261	CAMTA1	2,672955021
140766	ADAMTS14	-4,872994137
3887	KRT81	-6,386878974
552891	DNAJC25-GNG10	-6,707025199
55747	-	-9,399554375
3207	HOXA11	-4,546697163
84219	WDR24	-9,368695416
4322	MMP13	-6,371463567
6356	CCL11	-7,739447215
100463285	MTRNR2L4	-6,512645249
642778	NPIPA3	-5,411728297
4610	MYCL	3,225322468
467	ATF3	2,595210045
100529241	HSPE1-MOB4	-4,87121033
3481	IGF2	2,686929391

100529257	SYNJ2BP-COX16	-5,555238495
6490	PMEL	2,800604113
29113	C6orf15	-9,273536108
79840	NHEJ1	2,858927688
4046	LSP1	-6,31911874
7412	VCAM1	3,041787586
100526767	RNF103-CHMP3	-5,914528223
7538	ZFP36	3,284324006
100616408	MIR5047	-9,149896792
56475	RPRM	4,091290682
5100	PCDH8	2,396628988
5083	PAX9	-6,019853926
388555	IGFL3	-8,967834502
54715	RBFOX1	3,042793474
64093	SMOC1	2,858044286
650	BMP2	2,949234406
389136	VGLL3	2,830252663
100134938	UPK3BL	2,520781653
100008587	RNA5-8S5	4,360551281
6352	CCL5	2,515728428
6913	TBX15	2,716198523
3977	LIFR	4,021925627
7021	TFAP2B	4,208587976
5521	PPP2R2B	2,712655187
2354	FOSB	5,156457962
1602	DACH1	2,609768782
9465	AKAP7	2,704902229
7102	TSPAN7	3,558730716
100190939	TPT1-AS1	2,707914731
54861	SNRK	2,983795741
81558	FAM117A	2,896709065
6781	STC1	2,995600041
6750	SST	2,861956554

9118	INA	4,297540701
107	ADCY1	2,769615232
23600	AMACR	3,126382137
27065	NSG1	3,75373301
55203	LGI2	3,095538804
3164	NR4A1	5,510159342
1620	BRINP1	3,63159534
7020	TFAP2A	3,051315091
2045	EPHA7	2,928911282
10365	KLF2	3,794234591
286527	TMSB15B	3,116750287
100129931	LOC100129931	3,316736962
1756	DMD	2,862517359
6092	ROBO2	3,371657184
9980	DOPEY2	3,110748155
55384	MEG3	5,288915832
55619	DOCK10	3,001491887
79605	PGBD5	3,663618615
3038	HAS3	3,114898241
163486	DENND1B	4,153994732
4744	NEFH	3,410125502
389073	C2orf80	3,494996196
100302692	FTX	5,45343972
9314	KLF4	4,734443834
1745	DLX1	3,165938891
7855	FZD5	3,978443647
2034	EPAS1	3,522494457
335	APOA1	3,605686178
84708	LNX1	3,293197985
23237	ARC	4,018471468
353174	ZACN	3,389219899
1501	CTNND2	3,362832658
100132062	LOC100132062	3,828378945

167410	LIX1	3,415462565
6658	SOX3	3,589961249
4488	MSX2	4,203890016
55089	SLC38A4	4,318538139
55268	ECHDC2	3,583472607
4908	NTF3	5,539812106
55790	CSGALNACT1	3,529600255
8339	HIST1H2BG	4,812207001
9421	HAND1	4,930477861
1496	CTNNA2	4,13245759
112970	KTI12	3,494921842
100303728	SLC25A5-AS1	3,651853517
8912	CACNA1H	3,84026505
1746	DLX2	3,759713298
100873954	SNRK-AS1	4,759877585
2334	AFF2	3,907436656
64881	PCDH20	4,140978097
3429	IFI27	4,347225566
5101	PCDH9	4,976919858
340665	CYP26C1	4,798415909
1959	EGR2	6,029389468
1364	CLDN4	4,179061935
138649	ANKRD19P	7,647577804
100463488	MTRNR2L10	5,837579524
999	CDH1	4,820927101
5915	RARB	4,485748956
730130	TMEM229A	3,949662171
6926	TBX3	4,014855775
4745	NELL1	6,138919647
138046	RALYL	4,319145642
57111	RAB25	4,190340375
100499405	LINC00987	8,20053748
5800	PTPRO	5,152819878

202020	TAPT1-AS1	6,926849892
56956	LHX9	4,981374127
7471	WNT1	4,877757748
84419	C15orf48	4,834936304
139728	PNCK	4,897429262
163404	PLPPR5	4,722714002
1463	NCAN	7,914949704
1960	EGR3	5,812985928
9052	GPRC5A	6,244896371
22874	PLEKHA6	4,677518854
389125	MUSTN1	6,83238865
54897	CASZ1	5,512151409
339479	BRINP3	6,510224617
6543	SLC8A2	8,651326189
64072	CDH23	5,777593715
84966	IGSF21	5,943019305
65999	LRRC61	5,737460705
100131187	TSTD1	5,980457116
22915	MMRN1	5,78518376
339488	TFAP2E	6,796048879
196385	DNAH10	6,31558744
100506428	CBR3-AS1	10,60606961
174	AFP	6,435019809
4070	TACSTD2	6,109022028
2568	GABRP	8,095828585
2895	GRID2	6,782347759
5733	PTGER3	7,529730206
8809	IL18R1	6,889657783
116	ADCYAP1	7,656421857
3670	ISL1	7,119399351
64478	CSMD1	7,100219408
25924	MYRIP	7,75472351
100462983	MTRNR2L3	11,22655666

1749	DLX5	7,873741756
55655	NLRP2	8,927308683
NR_026650	-	9,319672121
54944	LINC01521	9,355901638
100505573	INAFM2	10,21340804
101928378	PTOV1-AS2	9,494355604
101410538	MMP24-AS1	10,73788108
440416	CCDC144NL-AS1	9,28424575
103344718	HOTS	14,32918317
374920	C19orf68	9,07881795
101927374	LOC101927374	9,768597882
163702	IFNLR1	9,018200179
100846978	LINC00506	10,97799537
104326058	SMAD1-AS1	9,321364432
644139	PIRT	10,59035313
100463487	MTRNR2L9	16,20308508
100506603	LOC100506603	10,45481335
353511	PKD1P6	11,18084157
100101440	PMS2P7	9,11504365
101060376	TBC1D3L	10,1179677
728769	SCAMP1-AS1	8,93737382
442117	GALNTL6	10,10819772
101929579	LOC101929579	9,443461612
102288414	C11orf98	12,75707743
100128494	LOC100128494	11,08148344
554249	LOC554249	9,222794903
100507347	VIM-AS1	14,40715156
727751	LOC727751	10,18363538
101926898	LOC101926898	9,702172685
101928663	LOC101928663	10,6110248
9023	CH25H	11,23451809
4880	NPPC	9,715533064
100506119	LINC01503	9,777666281

100132815	IPO5P1	9,591522347
102724699	LOC102724699	11,52454172
10590	SCGN	9,562242424
100419583	LOC100419583	9,730894647
201853	LINC00504	10,81398155
102723508	KANTR	13,22539575
101929147	LOC101929147	9,556506055
100288162	LOC100288162	10,70498432
4118	MAL	10,12960501
104413891	SAPCD1-AS1	8,992938336
79679	VTCN1	9,477252324
25818	KLK5	10,027906
103021295	LOC103021295	9,018895621
729348	LOC729348	10,40779885
101060691	NUTM2B-AS1	10,03032228
104472717	LINC01224	9,06608919
102157402	AK6	10,9432474
100505621	C11orf72	9,249706057
100507602	TRIM52-AS1	9,947636938
100131017	ZNF316	9,712097051
100288152	LOC100288152	9,077483357
388394	RPRML	9,117643101
100506365	OTUD6B-AS1	9,835260919
101926889	LOC101926889	8,987974524
100996301	FOXD3-AS1	10,20579325
400794	LOC400794	10,86418614
101927796	LINC01441	8,983706193
100130964	LOC100130964	11,63560477
729218	LOC729218	9,118292233
103625681	LLPH-AS1	8,918117851
103695435	BBOX1-AS1	8,978710459
100128055	SMARCA5-AS1	10,13891172
101752399	STAG3L5P-PVRIG2P-PILRB	9,804534403

100861548	PINK1-AS	9,472690839
100129434	LOC100129434	11,56522111
401242	LINC01623	12,14950898
100129195	ZSCAN16-AS1	8,990813079
246	ALOX15	9,762382039
7475	WNT6	9,853309555
285463	CTBP1-AS	9,494355604
100652748	TIMM23B	9,323617763
84777	DLGAP1-AS2	9,461479447
104169670	TMEM5-AS1	9,50779464
100505758	PRMT5-AS1	10,31316648
101154753	PANDAR	10,76735685
NM_001303027	-	9,635264657

Supplementary Table 3. List of genes with significantly altered expression in BVVL MNs B2 compared to BVVL MNs in RNA sequencing (fold-change ≥2 and diverge probability ≥0.8).

GeneID	Gene Symbol	log2Ratio(BVVL B2/BVVL)
9961	MVP	2,403776
NR_026650	-	-9,319672
1973	EIF4A1	-5,347842
200879	LIPH	3,65547
6218	RPS17	-6,736279
1843	DUSP1	-3,349839
7111	TMOD1	-2,481063
89	ACTN3	-2,928239
54944	LINC01521	-9,355902
100533975	SLMO2-ATP5E	-5,766343
29114	TAGLN3	-2,764192
100505573	INAFM2	-10,213408
79625	NDNF	-3,532392
7262	PHLDA2	2,764245
101928378	PTOV1-AS2	-9,494356
260436	FDCSP	8,850364
1018	CDK3	-6,526985
59284	CACNG7	-2,724887
9241	NOG	-2,766305
3880	KRT19	-4,472748
1535	CYBA	1,548725
1271	CNTFR	-2,150302
100534595	HNRNPUL2-BSCL2	-4,139189
5476	CTSA	2,063259
4191	MDH2	1,592713
5354	PLP1	-4,256153
63915	BLOC1S5	-4,819519
28984	RGCC	-3,900536
51652	CHMP3	-3,840997
23220	DTX4	-3,684456

5502	PPP1R1A	-3,585388
60482	SLC5A7	4,60601
58189	WFDC1	3,383053
92162	TMEM88	-2,114781
84973	SNHG7	-1,600348
100008588	RNA18S5	-16,286337
5121	PCP4	2,798583
81493	SYNC	-5,171966
100462977	MTRNR2L1	3,368861
93099	DMKN	-3,548625
10457	GPNMB	2,225328
6189	RPS3A	-2,285126
101410538	MMP24-AS1	-10,737881
54873	PALMD	4,538952
726	CAPN5	2,190846
1909	EDNRA	-3,245021
4804	NGFR	-2,716186
9636	ISG15	4,59647
80864	EGFL8	-6,258497
55240	STEAP3	2,052554
100129931	LOC100129931	-7,916356
199990	FAAP20	-2,001137
2969	GTF2I	-1,980813
1000	CDH2	-1,794145
3955	LFNG	-3,382148
84992	PIGY	-12,694467
8370	HIST2H4A	-9,316282
5441	POLR2L	1,869113
57584	ARHGAP21	1,670128
51015	ISOC1	1,67598
10346	TRIM22	2,079655
132228	LSMEM2	-5,12896
2681	GGTA1P	4,267405

7335	UBE2V1	-2,768256
1959	EGR2	-4,724287
100133286	LOC100133286	-4,799653
6235	RPS29	-1,987381
54843	SYTL2	-2,956777
6892	TAPBP	3,032562
440416	CCDC144NL-AS1	-9,284246
353174	ZACN	-7,909492
103344718	HOTS	-14,329183
80024	SLC8B1	2,33489
102606465	LOC102606465	-8,800091
11274	USP18	2,913929
83604	TMEM47	-2,398514
283070	-	10,159297
85477	SCIN	4,820956
6423	SFRP2	-3,427913
23237	ARC	-5,483852
7040	TGFB1	2,9082
948	CD36	4,722996
139728	PNCK	-4,908735
374920	C19orf68	-9,078818
6297	SALL2	-1,778142
5788	PTPRC	4,856814
94030	LRRC4B	-3,048388
9249	DHRS3	4,058986
84141	EVA1A	4,404472
57595	PDZD4	-3,011632
378938	MALAT1	4,66561
100137049	PLA2G4B	-5,668897
387885	CFAP73	-7,041027
101927374	LOC101927374	-9,768598
56829	ZC3HAV1	2,332265
6772	STAT1	1,738522

6173	RPL36A	-2,602384
NR_123729	-	-8,701306
54715	RBFOX1	-2,013141
84219	WDR24	9,800334
5721	PSME2	2,250889
6288	SAA1	4,377134
23217	ZFR2	-4,112013
1375	CPT1B	-2,482188
200030	NBPF11	-7,102583
117581	TWIST2	3,091844
4811	NID1	1,669171
128414	NKAIN4	-3,311024
51296	SLC15A3	2,326279
7546	ZIC2	-2,088169
4938	OAS1	6,949167
56896	DPYSL5	-1,89418
6347	CCL2	4,233642
85441	HELZ2	2,876907
2354	FOSB	-5,576539
5100	PCDH8	-2,337651
648	BMI1	-2,639341
5320	PLA2G2A	2,852758
51191	HERC5	4,793534
1316	KLF6	-1,755654
4072	EPCAM	-2,660952
112970	KTI12	-8,085233
114904	C1QTNF6	1,91421
55384	MEG3	-3,956718
10791	VAMP5	1,794516
29970	SCHIP1	-5,708589
10437	IFI30	4,060475
5764	PTN	-3,273175
51252	FAM178B	3,076803

597	BCL2A1	6,220379
100846978	LINC00506	-10,977995
100302652	GPR75-ASB3	-4,348204
245973	ATP6V1C2	-5,926897
100534599	ISY1-RAB43	-5,143196
6474	SHOX2	2,601753
55220	KLHDC8A	-3,734171
629	CFB	3,655996
104326058	SMAD1-AS1	-9,321364
100996928	C7orf55-LUC7L2	-7,558207
284889	MIF-AS1	-3,836282
10365	KLF2	-2,508831
378706	RN7SL2	2,950643
317762	CCDC85C	-2,083138
54541	DDIT4	-1,836587
26024	PTCD1	-6,201927
100463487	MTRNR2L9	-16,203085
4792	NFKBIA	2,059094
81567	TXNDC5	-3,627238
6700	SPRR2A	7,334723
3315	HSPB1	1,722369
5918	RARRES1	3,80559
55750	AGK	1,903456
644714	LIMD1-AS1	-6,71154
3732	CD82	-1,921829
586	BCAT1	-2,005644
50486	G0S2	3,163531
283820	NOMO2	-1,93361
7162	TPBG	-2,291508
100506603	LOC100506603	-10,454813
84933	C8orf76	-2,334553
57829	ZP4	2,824445
387597	ILDR2	-5,498934

23213	SULF1	3,282918
64135	IFIH1	5,937452
353511	PKD1P6	-11,180842
3800	KIF5C	-2,784261
1036	CDO1	-3,293261
7855	FZD5	1,973131
64093	SMOC1	-4,058711
3107	HLA-C	2,563957
1949	EFNB3	-2,027643
3791	KDR	3,618222
23304	UBR2	2,039758
5318	PKP2	-3,635008
9448	MAP4K4	1,876636
3398	ID2	-1,7543
567	B2M	3,608688
401251	SAPCD1	-8,769838
26579	MYEOV	6,406419
10561	IFI44	5,786873
4741	NEFM	-1,938815
100008589	RNA28S5	-17,948665
100101440	PMS2P7	-9,115044
101060376	TBC1D3L	-10,117968
3133	HLA-E	2,154227
100861402	CERS6-AS1	-8,272034
25927	CNRIP1	2,236451
136227	COL26A1	-2,375949
728769	SCAMP1-AS1	-8,937374
124976	SPNS2	-5,038268
102800317	LOC400927-CSNK1E	-8,762382
3188	HNRNPH2	-2,675782
100093631	GTF2IP4	-4,039647
6233	RPS27A	-1,537883
2274	FHL2	2,404199

2045	EPHA7	-2,259289
57533	TBC1D14	-1,938188
79132	DHX58	5,965917
10866	HCP5	4,482615
653247	PRB2	5,220556
4613	MYCN	-2,160392
100532731	COMMD3-BMI1	-5,189005
3665	IRF7	3,621644
8848	TSC22D1	-2,183991
10516	FBLN5	2,929128
3428	IFI16	3,287083
6490	PMEL	-2,155267
81848	SPRY4	2,017166
171019	ADAMTS19	-4,557624
10912	GADD45G	-2,497857
140597	TCEAL2	-2,993905
58498	MYL7	-3,575905
467	ATF3	-1,938822
11177	BAZ1A	1,734288
4831	NME2	-1,616486
145845	LOC145845	-8,015409
94240	EPSTI1	3,903703
8106	PABPN1	-2,005983
10581	IFITM2	2,319598
6658	SOX3	-3,931678
837	CASP4	4,113203
79008	SLX1B	-11,128317
6774	STAT3	1,748515
6206	RPS12	-1,546973
25791	NGEF	2,947489
8807	IL18RAP	3,545563
10150	MBNL2	2,355055
716	C1S	3,595198

101929579	LOC101929579	-9,443462
2014	EMP3	1,587956
723790	HIST2H2AA4	-10,791366
284618	RUSC1-AS1	-3,66647
3553	IL1B	8,776761
2022	ENG	2,18984
8363	HIST1H4J	-3,524786
6484	ST3GAL4	2,005557
102288414	C11orf98	-12,757077
100128494	LOC100128494	-11,081483
100134934	TEN1	-10,636851
5698	PSMB9	8,234866
199713	NLRP7	4,440091
347475	CCDC160	-2,857136
79953	SYNDIG1	1,979085
9353	SLIT2	2,093924
79856	SNX22	-4,486246
57687	VAT1L	-4,175576
51050	PI15	4,273174
7919	DDX39B	-3,944068
6695	SPOCK1	2,225307
4939	OAS2	7,985459
767558	LUZP6	-12,386267
8737	RIPK1	2,363226
286527	TMSB15B	-2,079965
124944	C17orf49	-2,770885
100506474	LOC100506474	4,76755
4326	MMP17	1,803101
9518	GDF15	4,15664
55601	DDX60	3,750961
56670	SUCNR1	2,337155
55008	HERC6	5,865419
249	ALPL	-2,11167

440926	H3F3AP4	-4,002477
5355	PLP2	2,069078
112398	EGLN2	-3,523498
145553	MDP1	-2,277178
1756	DMD	-2,234152
114814	GNRHR2	-3,740127
23780	APOL2	3,290954
23302	WSCD1	-3,393237
554249	LOC554249	-9,222795
6672	SP100	4,628443
3956	LGALS1	2,595272
2012	EMP1	2,831585
55969	C20orf24	-2,441681
100507347	VIM-AS1	-14,407152
8788	DLK1	-2,358103
6606	SMN1	-2,095238
101927746	LOC101927746	-8,668885
9843	HEPH	-2,267376
2633	GBP1	7,440358
2790	GNG10	-3,338361
1958	EGR1	-3,821955
29780	PARVB	2,290519
51540	SCLY	-3,561918
727751	LOC727751	-10,183635
101926898	LOC101926898	-9,702173
112616	CMTM7	1,600014
574042	SNORA10	5,401663
692158	SNORA57	5,066564
6422	SFRP1	-2,390303
101929074	PIK3CD-AS2	-8,881114
53916	RAB4B	-9,433462
138046	RALYL	-2,927219
101928663	LOC101928663	-10,611025

654483	BOLA2B	-11,405673
12	SERPINA3	7,528357
57633	LRRN1	-2,507644
5992	RFX4	-2,779182
79081	LBHD1	4,032308
9445	ITM2B	1,535578
653689	GSTT2B	-6,52023
7076	TIMP1	2,71667
23090	ZNF423	-2,070386
3178	HNRNPA1	-1,558393
115265	DDIT4L	3,615004
3669	ISG20	5,768445
56963	RGMA	-2,10201
9528	TMEM59	1,67351
100506119	LINC01503	-9,777666
100132815	IPO5P1	-9,591522
8651	SOCS1	2,433701
57194	ATP10A	3,385445
100529261	CHURC1-FNTB	-7,914552
100131205	RPL21P28	2,059287
10653	SPINT2	-2,147894
114769	CARD16	5,095071
102724699	LOC102724699	-11,524542
6775	STAT4	4,146887
11013	TMSB15A	-2,358747
100134938	UPK3BL	-3,855671
90326	THAP3	-2,050704
151636	DTX3L	4,142291
2568	GABRP	-7,868666
389073	C2orf80	1,798072
7178	TPT1	1,835729
100419583	LOC100419583	-9,730895
26580	BSCL2	-2,42345

7130	TNFAIP6	4,027131
1592	CYP26A1	2,44465
126328	NDUFA11	-1,567895
5413	SEPT5	-4,729317
24138	IFIT5	2,036204
201853	LINC00504	-10,813982
174	AFP	-3,527477
10123	ARL4C	-2,255472
6139	RPL17	-1,786243
163486	DENND1B	-3,390341
6515	SLC2A3	-2,554337
6237	RRAS	1,715122
51131	PHF11	2,301532
120237	DBX1	-7,246778
5936	RBM4	-2,238184
9695	EDEM1	2,951097
1364	CLDN4	-3,629043
151230	KLHL23	-6,048275
23559	WBP1	-3,474082
684	BST2	4,403953
7127	TNFAIP2	2,548532
6769	STAC	3,947144
126868	MAB21L3	3,295627
999	CDH1	-4,020208
4599	MX1	6,744828
9890	PLPPR4	1,813674
102723508	KANTR	-13,225396
83666	PARP9	3,721344
5359	PLSCR1	3,151582
84220	RGPD5	-4,956986
129607	CMPK2	4,369614
167826	OLIG3	-2,837107
388152	GOLGA2P7	-2,519328

101929147	LOC101929147	-9,556506
100288162	LOC100288162	-10,704984
57216	VANGL2	-1,711482
100302692	FTX	-6,084344
3020	H3F3A	-3,965134
7276	TTR	-3,83083
1404	HAPLN1	-2,916899
80755	AARSD1	-4,580722
2619	GAS1	-2,183381
6477	SIAH1	-2,287096
7044	LEFTY2	3,044349
4062	LY6H	-5,106483
1280	COL2A1	-2,159575
6876	TAGLN	-3,382011
7980	TFPI2	7,246089
6607	SMN2	-2,27157
100526694	MSANTD3-TMEFF1	-6,347594
23645	PPP1R15A	-1,585767
347733	TUBB2B	-2,766179
692224	FBXO22-AS1	-10,107016
4118	MAL	1,759306
390	RND3	-1,664608
1515	CTSV	-3,576109
104413891	SAPCD1-AS1	-8,992938
3725	JUN	-3,145091
84171	LOXL4	3,63024
10630	PDPN	1,802589
388	RHOB	-2,841365
9180	OSMR	2,496183
401138	AMTN	3,204745
6029	RN7SL1	3,596719
1075	CTSC	-1,725949
171425	CLYBL	-2,854928

401944	LDLRAD2	-4,690769
10133	OPTN	1,726562
10964	IFI44L	6,296332
4055	LTBR	2,655651
56603	CYP26B1	2,411061
5634	PRPS2	1,856906
8926	SNURF	-5,361119
131405	TRIM71	-3,556446
6524	SLC5A2	-6,020772
9976	CLEC2B	5,966409
5602	MAPK10	-4,282542
5098	PCDHGC3	-2,898711
388524	RPSAP58	-1,833157
3959	LGALS3BP	3,017845
6634	SNRPD3	1,713383
100190939	TPT1-AS1	-2,213057
54809	SAMD9	5,792475
25878	MXRA5	2,415149
25939	SAMHD1	3,33443
3106	HLA-B	4,196043
79679	VTCN1	-8,171419
8577	TMEFF1	-4,394266
2034	EPAS1	-5,068316
64761	PARP12	4,453409
8519	IFITM1	5,722491
91461	PKDCC	-3,573762
1890	TYMP	4,157161
4681	NBL1	-3,959075
8324	FZD7	-1,81996
3489	IGFBP6	2,95155
90050	FAM181A	-6,301432
51513	ETV7	6,202893
6398	SECTM1	2,21416

3430	IFI35	6,269871
400916	CHCHD10	2,069313
100271927	RASA4B	-4,765233
8337	HIST2H2AA3	-10,791366
92369	SPSB4	2,133346
80154	GOLGA2P10	-2,708614
92293	TMEM132C	-3,650653
1400	CRMP1	-1,620692
103021295	LOC103021295	-9,018896
635	BHMT	5,597346
3875	KRT18	-1,622977
94239	H2AFV	-1,636084
341640	FREM2	-3,981074
3672	ITGA1	2,365111
9592	IER2	-2,663636
114984	FLYWCH2	1,651202
4110	MAGEA11	2,247265
3576	CXCL8	2,957917
729348	LOC729348	-10,407799
624	BDKRB2	4,264922
7453	WARS	1,845063
151195	CCNYL1	1,980044
6227	RPS21	-1,967522
23779	ARHGAP8	-4,253004
2073	ERCC5	-3,058802
150094	SIK1	-3,003239
101060691	NUTM2B-AS1	-10,030322
2118	ETV4	1,831974
1153	CIRBP	-1,658558
762	CA4	3,644237
5281	PIGF	-2,2774
874	CBR3	3,296767
202020	TAPT1-AS1	-6,585008

1026	CDKN1A	2,265842
1469	CST1	-4,261129
79570	NKAIN1	-2,269961
6519	SLC3A1	-7,504968
6737	TRIM21	1,994063
286204	CRB2	-3,455098
4070	TACSTD2	-6,749994
761	CA3	-2,96991
83543	AIF1L	-2,373119
201780	SLC10A4	2,62421
5260	PHKG1	-4,101831
7471	WNT1	-5,806407
79825	EFCC1	-5,943101
91653	BOC	-2,260439
6781	STC1	-2,645292
100499405	LINC00987	-8,359963
23261	CAMTA1	-2,829369
124641	OVCA2	-2,146078
3429	IFI27	6,197897
1992	SERPINB1	2,438243
389125	MUSTN1	-3,849662
104472717	LINC01224	-9,066089
10379	IRF9	3,143155
57111	RAB25	-3,525457
1420	CRYGC	9,302957
3437	IFIT3	5,842473
677805	SNORA18	10,663274
6890	TAP1	4,448698
3400	ID4	-2,763842
102157402	AK6	-10,943247
5641	LGMN	1,64658
8638	OASL	8,326284
3910	LAMA4	1,65938

857	CAV1	2,129229
554313	HIST2H4B	-9,316282
145741	C2CD4A	2,897606
100506428	CBR3-AS1	-7,011826
618	BCYRN1	7,758791
6023	RMRP	6,021285
22915	MMRN1	-5,505762
30851	TAX1BP3	-2,714261
29907	SNX15	-5,815621
388125	C2CD4B	4,293066
6160	RPL31	-2,514006
5292	PIM1	-2,049791
4940	OAS3	6,075001
477	ATP1A2	-2,947311
6663	SOX10	-5,483666
8542	APOL1	4,682304
6229	RPS24	-2,491896
1463	NCAN	-2,007647
1029	CDKN2A	2,194764
11165	NUDT3	-2,364047
730394	GTF2H2C_2	-2,503149
6580	SLC22A1	6,747403
1947	EFNB1	-1,826817
6654	SOS1	1,833948
129080	EMID1	-2,367219
1052	CEBPD	2,662263
100505621	C11orf72	-9,249706
100507602	TRIM52-AS1	-9,947637
290	ANPEP	2,978641
100131017	ZNF316	-9,712097
25825	BACE2	1,952295
283659	PRTG	-2,806193
100288152	LOC100288152	-9,077483

8404	SPARCL1	2,550766
29057	FAM156A	-3,071577
7025	NR2F1	-3,105622
6549	SLC9A2	5,933352
81788	NUAK2	-3,308658
100507424	LOC100507424	-3,275037
2649	NR6A1	-4,433
9829	DNAJC6	2,092385
1960	EGR3	-4,189412
390992	HES3	-3,486053
3569	IL6	4,154347
10519	CIB1	1,619558
5420	PODXL	-2,838553
84163	GTF2IRD2	-4,99046
10148	EBI3	4,341349
25925	ZNF521	-2,656231
9421	HAND1	-5,951421
3856	KRT8	-2,971037
101928649	CTC-338M12.4	-8,704768
3897	L1CAM	-3,820273
6657	SOX2	-2,120856
1381	CRABP1	-2,906016
54437	SEMA5B	-4,155146
83690	CRISPLD1	1,562102
81557	MAGED4B	-7,613836
5652	PRSS8	-2,822517
9118	INA	-1,903143
441381	LRRC24	-3,932548
1373	CPS1	2,062607
388394	RPRML	-4,409391
3490	IGFBP7	2,309598
100529063	BCL2L2-PABPN1	-8,580946
11217	AKAP2	-7,471955

4696	NDUFA3	1,577589
79727	LIN28A	-1,797211
8663	EIF3C	-4,379989
100506365	OTUD6B-AS1	-9,835261
84216	TMEM117	3,197562
4008	LMO7	2,622057
402665	IGLON5	-2,954641
7280	TUBB2A	-2,285365
8536	CAMK1	1,917992
358	AQP1	-3,381048
60489	APOBEC3G	2,843896
94009	SERHL	-5,476333
6204	RPS10	-1,911872
158960	CH17-340M24.3	-8,642954
25843	MOB4	-3,427906
100873965	MED4-AS1	-5,850481
114928	GPRASP2	-4,112089
57169	ZNFX1	2,711711
4061	LY6E	2,165414
2537	IFI6	5,620465
55337	C19orf66	4,025095
9111	NMI	3,133762
101926889	LOC101926889	-8,987975
5998	RGS3	-2,022334
55861	DBNDD2	-3,653646
2558	GABRA5	4,477427
100996301	FOXD3-AS1	-10,205793
445329	SULT1A4	-6,624207
3164	NR4A1	-4,742144
100873954	SNRK-AS1	-6,83671
100008587	RNA5-8S5	-13,048998
3431	SP110	3,880479
400794	LOC400794	-10,212101

100302739	PCNA-AS1	-9,885162
3627	CXCL10	7,091449
10447	FAM3C	1,700942
652	BMP4	-2,426614
101928062	LINC01481	-8,795228
440823	MIAT	-2,885952
58472	SQRDL	3,718721
1001	CDH3	-3,187117
10537	UBD	6,643581
101927796	LINC01441	-8,983706
554282	FAM72C	-8,703904
100170229	SRRM5	-4,712937
100132062	LOC100132062	-4,289659
441454	LOC441454	4,74257
563	AZGP1	4,293495
57412	AS3MT	-4,147029
7164	TPD52L1	3,182463
2180	ACSL1	2,379191
5696	PSMB8	5,356934
55843	ARHGAP15	3,333521
80712	ESX1	4,381949
729218	LOC729218	-9,118292
84969	TOX2	2,248104
728492	SERF1B	-6,812252
55531	ELMOD1	1,748797
103625681	LLPH-AS1	-8,918118
1374	CPT1A	3,057205
103695435	BBOX1-AS1	-8,97871
4862	NPAS2	3,425746
654364	NME1-NME2	-3,233889
729438	GATSL2	-7,054841
100128055	SMARCA5-AS1	-10,138912
9074	CLDN6	-2,376995

7318	UBA7	3,543325
7832	BTG2	-1,940943
6656	SOX1	-6,827219
1490	CTGF	-3,254859
81894	SLC25A28	2,075732
25764	HYPK	-6,510652
4675	NAP1L3	1,984447
101752399	STAG3L5P-PVRIG2P-PILRB	-9,804534
9830	TRIM14	3,945601
100507118	PRC1-AS1	-3,547136
389658	FAM150A	5,173619
100750247	HIF1A-AS2	-8,416955
728239	MAGED4	-5,937046
4782	NFIC	1,895285
154791	FMC1	-2,150518
404665	CACTIN-AS1	-4,324216
10874	NMU	1,999991
51083	GAL	2,218875
1728	NQO1	1,694718
220869	CBWD5	-3,460367
27319	BHLHE22	2,453934
6750	SST	-4,330637
130733	TMEM178A	1,723609
101927027	LOC101927027	-8,686501
5266	PI3	7,140282
3481	IGF2	-4,180004
26292	MYCBP	-2,868599
162394	SLFN5	2,427618
90525	SHF	-2,100386
602	BCL3	2,92484
57348	TTYH1	-2,037325
387680	WASHC2A	-3,211832
100526830	SLX1A-SULT1A3	-5,123161

4502	MT2A	2,591576
4666	NACA	-2,543413
780851	SNORD3A	2,972243
53826	FXYD6	-1,93588
100861548	PINK1-AS	-9,472691
10439	OLFM1	-3,628722
2261	FGFR3	-2,691608
6638	SNRPN	-6,280435
3785	KCNQ2	-4,143675
715	C1R	3,443558
23117	NPIPB3	-2,897193
23224	SYNE2	-2,666906
100526737	RBM14-RBM4	-4,892469
677822	SNORA40	9,230623
654320	SNORA8	6,253772
8809	IL18R1	2,11343
100462983	MTRNR2L3	-9,440644
3055	HCK	3,447397
100129434	LOC100129434	-11,565221
100874222	RNF219-AS1	-8,243075
3434	IFIT1	4,169332
7268	TTC4	-2,615057
401242	LINC01623	-11,155409
100129195	ZSCAN16-AS1	-8,990813
9997	SCO2	-3,428346
4060	LUM	-5,949967
246	ALOX15	1,901169
5610	EIF2AK2	2,366487
3241	HPCAL1	1,616548
54795	TRPM4	2,268637
84875	PARP10	3,971632
796	CALCA	5,14102
2733	GLE1	1,772384

57048	PLSCR3	-2,483513
100463488	MTRNR2L10	-6,875094
100861532	RNA45S5	2,066891
79583	TMEM231	-4,760446
9211	LGI1	-2,452381
6891	TAP2	2,594136
138649	ANKRD19P	-7,986238
84154	RPF2	1,836614
3988	LIPA	2,083287
9580	SOX13	-2,270484
11211	FZD10	-5,800205
285463	CTBP1-AS	-9,494356
2048	EPHB2	-3,020208
84632	AFAP1L2	2,552559
100652748	TIMM23B	-9,323618
100303728	SLC25A5-AS1	-3,76168
548593	SLX1A	-11,128317
54625	PARP14	3,559314
375295	LINC01116	3,198211
3383	ICAM1	4,846643
2970	GTF2IP1	-4,211454
100529097	RPL36A-HNRNPH2	-4,883322
100507577	LOC100507577	-2,865746
84777	DLGAP1-AS2	-9,461479
4907	NT5E	5,487778
10410	IFITM3	3,677345
7070	THY1	2,30932
56521	DNAJC12	2,319753
7538	ZFP36	-1,78519
4744	NEFH	2,366939
11166	SOX21	-5,869803
10468	FST	-2,189431
27022	FOXD3	-5,108014

5205	ATP8B1	4,197005
95	ACY1	-2,865205
8293	SERF1A	-7,104754
7903	ST8SIA4	3,374392
340665	CYP26C1	-7,186233
7168	TPM1	-2,349985
51631	LUC7L2	-2,191251
85495	RPPH1	3,286407
81578	COL21A1	2,676149
1471	CST3	1,878789
84419	C15orf48	3,371432
7697	ZNF138	-2,546403
6590	SLPI	5,729274
5920	RARRES3	8,98355
4600	MX2	6,566646
100532737	ATP6V1G2-DDX39B	-5,882481
4488	MSX2	-2,886209
2353	FOS	-4,695014
3433	IFIT2	5,139303
104169670	TMEM5-AS1	-9,507795
101928600	LOC101928600	-8,660887
23600	AMACR	-3,268195
100506963	TCEB3-AS1	-3,728933
100505758	PRMT5-AS1	-10,313166
5269	SERPINB6	2,110446
552900	BOLA2	-4,014941
3417	IDH1	1,920174
1500	CTNND1	-3,989175
55502	HES6	-1,865207
125050	RN7SK	3,093353
7846	TUBA1A	-1,710153
9235	IL32	4,446247
23586	DDX58	3,638675

3491	CYR61	-1,914295
54704	PDP1	1,654288
101154753	PANDAR	-10,767357
10810	WASF3	-2,641364
NM_001303027	-	-9,635265
54739	XAF1	7,332695
7545	ZIC1	-6,62909
10239	AP3S2	-3,019433
79630	C1orf54	-2,290659
